# Defect-Engineered High-Entropy Spinel Oxide@Onion-Like Carbon Catalysts for High-Areal-Energy Rechargeable Zinc–Air Batteries

**DOI:** 10.1021/acs.energyfuels.5c02012

**Published:** 2025-06-25

**Authors:** Agnes Mongwe, Aderemi B. Haruna, Lesego Gaolatlhe, Joesene Soto, Zixiao Shi, Patrick V. Mwonga, Xiao-Yu Yang, David A. Muller, Héctor D. Abruña, Kenneth I. Ozoemena

**Affiliations:** † School of Chemistry, Molecular Sciences Institute, 37707 University of the Witwatersrand, PO Wits, Johannesburg 2050, South Africa; ‡ Department of Chemistry and Chemical Biology, 5922 Cornell University, Ithaca, New York 14850, United States; § Kavli Institute at Cornell for Nanoscale Science, Cornell University, Ithaca, New York 14850, United States; ∥ School of Applied and Engineering Physics, Cornell University, Ithaca, New York 14850, United States; ⊥ State Key Laboratory of Advanced Technology for Materials Synthesis and Processing, School of Materials Science and Engineering, 12565 Wuhan University of Technology, Wuhan 430070, China

## Abstract

Rechargeable zinc-air batteries (ReZAB) have emerged as the next-generation batteries with several advantages over the conventional lithium-ion battery. In this work, single nanocrystals of inverse-type high-entropy spinel oxides (HESOx, particle size of 10–12 nm) confined in highly curved defective onion-like carbons (HESOx/OLC_AT_) as efficient electrocatalysts for oxygen evolution reaction (OER), oxygen reduction reaction (ORR), and ReZAB, have been synthesized. The HESOx materials were thoroughly characterized using several analytical techniques, including X-ray diffraction (XRD), X-ray photoelectron spectroscopy (XPS), scanning transmission electron microscopy (STEM), Raman, and electron paramagnetic resonance (EPR). HESOx/OLC_AT_ catalyst was tested for ReZAB using literature-recommended parameters that would allow for real technological application. These parameters include a current loading of 10 mA cm^–2^ and a discharge areal energy density of 35 mWh cm_geometric_^–2^, which maps a Li-ion battery pack-level specific energy of 120 Wh kg_pack_^–1^. HESOx/OLC_AT_ electrocatalysts allowed for continuous discharging and charging at a current loading of 10 mA cm^–2^ with discharge areal energy densities between 37 and 74 mWh cm_geometric_^–2^, thus outperforming the recommended threshold of 35 mWh cm_geometric_^–2^. Considering that most studies (>90%) hardly meet the recommended threshold for technological application of ReZAB, the present work represents one of the top-performing electrocatalysts for ReZAB. The excellent electrocatalytic properties of defect-rich HESOx/OLC_AT_ toward ORR/OER and ReZAB are governed by the strong electronic modulation arising from d-π hybridization, the availability of multiple catalytic sites for intermediates, and weakened d-band centers of the rate-determining intermediates (i.e., *O adsorption for ORR and *OOH formation for OER) compared to the pristine HESOx. This work introduces an effective approach for the design and synthesis of single nanocrystals of high-entropy electrocatalysts for the development of low-cost, robust, and technologically relevant rechargeable zinc–air batteries.

## Graphical abstract

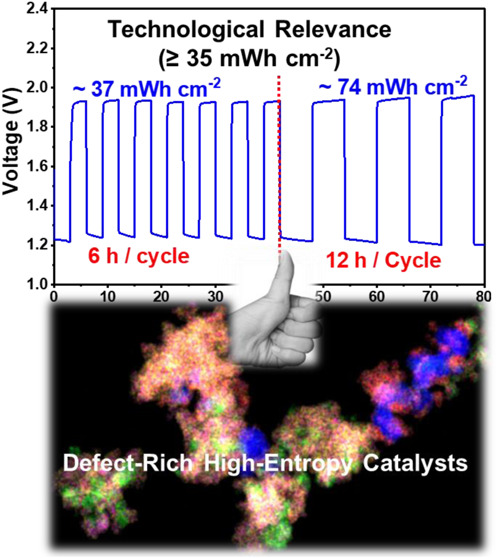

## Introduction

Energy storage is still one of the most important challenges facing the global economy. It is critical if the world must fulfill some of its most urgent sustainable development goals, such as good health and well-being, affordable and clean energy, and mitigation of climate deterioration, to mention only a few. It is generally accepted that battery technologies are a key part of the puzzle when it comes to enabling widespread electric transportation, personal electronics, smart grids/cities, renewable energy, and off-grid electrification. Global commitment to reduce emissions in the transport sector has led to the emergence of new energy vehicles (NEVs) with a smaller carbon footprint. Battery electric vehicles (BEVs) are gaining traction because of their low emission profile.

For more than three decades, lithium-ion batteries (LIBs) have dominated the battery industry. However, aside from being too expensive and unsafe, it has been accepted that LIBs are fast approaching their maximum ability to store and generate electricity. Therefore, there is an urgent need for advanced batteries that can meet the energy demand of the next generation of consumer technologies. Rechargeable zinc-air battery (ReZAB) is a battery technology with a huge potential to meet the Sustainable Development Goal (especially, Goal # 7: Affordable and clean energy) of the United Nations. ReZAB is a safe and environmentally friendly technology. ReZAB can provide a localized energy source for powering electronic devices, electric vehicles, and power reserves while improving access, safety, and reliability. ReZAB represents one of the unique battery technologies, as it uses ordinary air (oxygen), cheap zinc, and alkaline water to store and generate electricity.

ReZAB boasts several advantages over the LIB and other battery technologies,^1–3^ and has been gaining traction in recent years.^4–7^ For example, (1) it is safe to use (it uses water as an electrolyte, not the flammable organic liquid used in LIB); (2) it is a low-cost technology because of the natural abundance of its critical raw materials (zinc, air, water, and transition metals such as manganese). In general, unlike other battery technologies, such as LIBs, lead-acid batteries, and nickel–metal hydride, ReZAB is environmentally benign and recyclable. Additionally, the abundance of its materials does not constitute the risk of low supply; (3) it possesses high theoretical specific energy density (1086 Wh/kg), which is about five times greater than that of the conventional LIB; (4) the practical specific energy density of ReZAB is the same as that of the Li–air battery (450 wh/kg); and (5) its manufacturing/capital cost (about US$50) is as low as that of the conventional lead-acid battery.

For these compelling reasons, ReZAB can be used in a plethora of applications, such as portable electronics (such as mobile phones and laptops), wearable power electronics, grid electricity storage, home electricity, electric vehicles, and aircraft, to mention a few. Despite the huge advantages of ReZABs, some technical challenges have continued to conspire against their widespread development and commercialization. A key challenge is the sluggish kinetics of the oxygen reduction reaction (ORR) and oxygen evolution reaction (OER). Although carbon-supported noble metal electrocatalysts such as platinum group metals (PGMs) can improve the kinetics of ORR, they are extremely poor for OER.^8,9^ The high cost and low electrochemical stability of PGMs make them undesirable for large-scale/practical applications. Thus, there is a continuing need to develop robust and low-cost transition-metal-based bifunctional electrocatalysts that will drive ORR and the OER in ReZABs.

In the past decade, there has been an increased interest in the R&D of ReZAB because of the urgent global demand for a more affordable, safe-to-use, environmentally friendly, and high-energy battery technology that can support the next generation of commercial and renewable energy technologies. There are several scientific papers on ReZABs that have claimed to have made some technological breakthroughs in this battery system. However, in 2020, Rolison group examined these scientific articles, and very disappointingly, found that only 8 out of the 100 articles could claim to have made a real technological breakthrough.^2^ According to Rolison and co-workers, a real technological breakthrough in ReZAB can be claimed only if the ReZAB can compete with lithium-ion batteries. According to the authors, “*to compete with Li-ion batteries, researchers should cycle zinc–air cells at 35 mWh cm* ^*–2*^”.^2^

Motivated by the proposed threshold for developing technologically relevant ReZABs, we report on defect-rich single nanocrystals of high-entropy inverse spinel oxides (CuMnFeNiCo)_3_O_4_ on onion-like carbons (OLC) (herein abbreviated as HESOx/OLC_AT_) as a highly efficient bifunctional electrocatalyst for ORR/OER and ReZAB. Corner and edge sites of catalysts have been known to exhibit enhanced electrocatalytic activities. To mimic metal sites at the corners and edges of particles, this work uses highly curved carbon support to anchor HESOx sites. The excellent physicochemical properties of OLC, including high-curvature, small nanoparticles <10 nm, and high electrical conductivity (∼4 S cm^–1^), make it the ultimate candidate for anchoring HESOx. We show that HEXOx/OLC_AT_ complex achieved 74 mWh cm_geometric_^–2^ (Note that ‘geometric’ means the geometric surface area of the electrode or current collector) exceeding the threshold of 35 mWh cm_geometric_^–2^ at a current loading of 10 mA cm^–2^ with excellent ‘bifunctionality index’ (Δ*E* = |*E*_1/2(ORR)_ – *E*_*j*=10(OER)_|) of 0.75 V. The effect of strong electronic modulation arising from d-π hybridization and weakened d-band centers in the HESOx/OLC_AT_ complex that allowed for the superior electrocatalysis for ReZAB are highlighted.

## Experimental Section

### Materials

The reagents used in this study were of analytical grade and were used without further purification. The precursors, Co­(NO_3_)_2_·6H_2_O, Cu­(NO_3_)_2_·3H_2_O, Fe­(NO_3_)_3_·9H_2_O, Mn­(NO_3_)_2_·4H_2_O, Ni­(NO_3_)_2_·6H_2_O, ethylene glycol, and citric acid were purchased from Sigma-Aldrich. The onion-like carbon (OLC) was obtained by annealing denoted nanodiamonds (98–99% purity, NaBond Technologies Co.) at a temperature of 1300 °C as described in ref 3. Commercial Pt/C (10 wt %) and IrO_2_ (99.9%) were also obtained from Sigma-Aldrich.

### Synthesis of Single Nanocrystals of High-Entropy Inverse Spinel Oxides (CoCuFeMnNi)_3_O_4_ (HESOx)

The high-entropy spinel oxides were synthesized using a one-step powder-forming Pechini method,^10^ exemplified in Figure S1. Briefly, stoichiometric amounts of the metal nitrate salts were prepared, where each metal nitrate salt was dissolved in deionized water and magnetically stirred to obtain adequate homogeneity. Citric acid was dissolved in deionized water, and this was followed by the addition of ethylene glycol to obtain the CA-EG mixture (1:4). The metal nitrate solutions were mixed and added dropwise into the CA-EG mixture and left to stir while heating at 90 °C. The metal nitrate solution eventually dehydrated into a gel but was kept at 90 °C until the gel burned, ultimately forming a porous black metal oxide powder. The powder was subsequently calcined at 500 °C for 6 h in air to obtain a black powder and subsequently annealed at 550 °C for 6 h under inert argon atmosphere to obtain a black crystalline powder (designated herein as **HESOx**).

### Loading HESOx onto Onion-like Carbon (OLC)

The loading of HESOx onto the OLC is summarized as shown in Figure S2. Briefly, 360 mg of OLC was first stirred in 20 mL of ethylene glycol in a 3-neck flask. Into this, “solution A” (containing 4 mL of water, 4 mL of ethylene glycol, 0.5 mL of HCl (32%), and 40 mg of HESOx) was added dropwise. A separate mixture of NaOH (0.40 g) in H_2_O (0.80 mL) mixed in EG (2.8 mL) was prepared (“Solution B”) and added to the mixture. The reaction mixture was heated at 125 °C for 3 h under a nitrogen atmosphere. After cooling to room temperature, the solid products were washed with distilled water until the filtrate pH became neutral. The final product was then dried at 40 °C in a vacuum oven (designated as **HESOx/OLC**_**AT**_, where “AT” signifies acid/alkaline treatment). The synthesis of **HESOx/OLC** follows the same procedure, except that HCl and NaOH were omitted in the sample preparation. The stoichiometry of the metal composition was established using inductively coupled plasma atomic emission spectroscopy (ICP-AES) at the Cornell Nutrient Analysis Laboratory (CNAL) using conventional digestion with aqua regia (HNO_3_/HCl 1:4 v/v).

### Physical Characterization

The scanning transmission electron microscopy (STEM) experiment was performed using Thermo Fisher FEI Spectra 300 TEM with ultrahigh-brightness cold field emission gun (C-FEG) and spherical aberration correction (aka. “Kraken” at Cornell University). The STEM resolution is 58 pm with probe current = 75 pA. The high-annular angle dark-field (HAADF) image was collected with C2 aperture = 70 μm, camera length = 94 mm, and convergence angle = 30 mrad with 60 mrad inner and 200 mrad out collection angles. The HAADF image stacks were taken with 2048 × 2048 pixels with 200 ns dwell times to 30 frames, and then the image stacks were summed to one frame after rigid registration to enhance signal-to-noise ratio (SNR) and reduce the effect of sample drift. The energy-dispersive X-ray spectroscopy (EDS) experiment was performed using a Super-X EDS detector with probe current = 600 pA.

XRD characterizations were performed on a Bruker D2 phaser. The Zeiss Auriga Field emission scanning microscopy (SEM) powered by SmartSEM was used to obtain SEM images, and the Oxford X-max with Aztech software was used to obtain EDX data. Thermogravimetric analyses (TGA) of the samples were carried out using a TGA 4000 Perkin Elmer at a heating rate of 10 °C/min, under air (20 mL/min) from 35 to 900 °C. The surface structure of the catalysts was examined using a Thermo Fisher Scientific Nexsa G2 Surface analysis system, which was equipped with an aluminum Kα source (1486.68 eV) from the Cornell Center for Materials Research (CCMR) at Cornell University. A spot size of 400 μm was selected for all of the measurements. The survey spectrum was recorded with a resolution/pass energy of 200 eV and an energy step size of 1 eV. The elemental regions were recorded with a resolution/pass energy of 50 eV and a step size of 0.1 eV to obtain better resolution. All of the samples were measured in powder form using copper tape as the loading template.

### Electrochemical Testing (Half-Cell Measurements)

The electrochemical measurements were carried out at room temperature using the Biologic VSP300 potentiostat powered by EC lab or Pine Research Instrumentation WaveDriver 200 potentiostat with a rotating disk electrode (RDE) setup. Briefly, for the HESOx, HESOx/OLC, and HESOx/OLC_AT_, each sample was dispersed in pure ethanol (1 mL) containing 5% Nafion (50 uL) and sonicated in ice-cold water for 1 h. Carbon rod or Pt rod was used as the counter electrode, while Ag|AgCl (3 M KCl) was used as the reference electrode. Oxygen-saturated 1 M KOH was used as the electrolyte for both the ORR and the OER. Linear sweep voltammograms (LSV) were conducted at a scan rate of 10 mV/s. The area of the electrode or current collector in this work is geometric rather than gravimetric or an electroactive surface area. The electrochemical impedance spectroscopy (EIS) was performed at a frequency range of 100 kHz to 0.01 Hz and *E*_*j*=10_ potential for different materials.

Rotating ring and disk electrode (RRDE) experiments were conducted with an electrode consisting of a glassy carbon disk and gold ring. The ORR activity was tested by using CV with an RRDE setup. Background measurements were run first, starting at 1.1 V and finishing at 0.4 V vs RHE at a scan rate of 5 mV s^–1^ and 1600 rpm in an Ar-saturated 1 M KOH. For the ORR measurements, an additional CV was acquired at the same potential window, scan rate, and rpm while under oxygen-saturated 1 M KOH solution. The RRDE experiments were done by selecting a bias of 1.2 V versus RHE at the ring in collection efficiency mode to acquire the current associated with peroxide formation.

Using the geometrical area of the electrode, the current density was normalized and the potentials were converted to a reversible hydrogen electrode (RHE) using the following Nernst equation

1 $$E_{\mathrm{RHE}}=E_{\mathrm{Ag}/\mathrm{AgCl}}+0.059\times \mathrm{pH}+0.1976$$

To calculate the overpotential (η) for the OER, the following formula was used

2 $$\eta \;\left(V\right)=E_{\mathrm{RHE}}-1.23$$

For the ORR, analysis was done using CV data taken at 2025, 1600, 1225, 900, 625, and 400 rpm. The limiting current values at 0.5 V of all of the CVs were used and the number of electrons transferred (*n*) from the RDE experiments was calculated using the Koutecký–Levich (KL) equation

3 $$\frac{1}{j}=\frac{1}{j_{k}}+\left(\frac{1}{0.62\mathrm{nFA}D_{o}^{2/3}\vartheta^{-1/6}C_{o}}\right)\omega^{-1/2}$$

where *j* is the current density, *j*_k_ is the kinetic current density, *F* is the Faraday constant (96 485.3321 C mol^–1^), *A* is the geometric area of the electrode, *D*_o_ is the diffusion coefficient of oxygen (1.93 × 10^–5^ cm^2^ s^–1^), υ is the viscosity of the solution (1.009 × 10^–2^ cm^2^ s^–1^), *C*_o_ is the concentration of oxygen dissolved in solution (0.78 × 10^–6^ mol cm^–3^), and ω is the rotation speed in rpm.

H_2_O_2_ selectivity and electron transfer numbers (*n*) were calculated from the RRDE experiments using the following equations

4 $$H_{2}O_{2}\;s\mathrm{electivity}:\;\%H_{2}O^{-}=200\left(\frac{\frac{I_{ring}}{N}}{I_{disk}+\frac{I_{ring}}{N}}\right)$$

5 $$\mathrm{electron}\;\mathrm{transfer}\;\mathrm{number}:\;n=4\left(\frac{4I_{\mathrm{disk}}}{I_{\mathrm{disk}}+\frac{I_{\mathrm{ring}}}{N}}\right)$$

where the polarization curves were used to calculate the Tafel slopes.

6 η=b(log⁡j)+a

where *b* is the Tafel slope and *j* is the disk current.

### Fabrication and Testing of Rechargeable Zinc–Air Battery (ReZAB) Cells

A homemade cell was fabricated to measure the performance of the catalysts in rechargeable zinc–air batteries. A zinc plate with a thickness of 0.25 mm was used as the anode, and the gas diffusion layer (carbon paper) coated with the catalyst was used as the air cathode. The carbon paper with a geometric area of 1 cm^2^ was used, and 10 mg of the catalyst was loaded on the paper. A conventional electrolyte (0.2 M zinc acetate dissolved in 6 M KOH) was used. To prepare the catalyst slurry, 10 mg of HESOx/OLC or HESOx/OLC_AT_ was dissolved in 20 μL of water, 180 μL of isopropyl alcohol, and 20 μL of PTFE and stirred on a magnetic stirrer. The coated catalyst was oven-dried at 80 °C for 30 min. A rechargeable zinc–air battery with a mixture of Pt/C and IrO_2_ (mass ratio of 1:1) was assembled in the same way for comparison. The biologic VSP300 potentiostat was used to collect the reported data on rechargeable zinc–air batteries. The long-term stability was first conducted under shallow cycling conditions at 2 mA cm^–2^ with 30 min discharge and 30 min charge (1 h per cycle). The catalyst was then subjected to harsh cycling conditions (6 and then 12 h per cycle) to meet the minimum required depth of discharge.

## Results and Discussion

### Physical Characteristics

ICP-AES analysis (Supporting Information) was used to estimate the stoichiometry of the HESOx/OLC_AT_ as (Cu_0.17_Mn_0.18_Fe_0.18_Ni_0.23_Co_0.24_)_3_O_4_, which is approximately the expected 0.6 per metallic ion. Figure S3 compares the transmission electron microscope (TEM) images of pristine HESOx, HESOx/OLC, and HESOx/OLC_AT_ at 50 nm resolution. The pristine HESOx comprises single nanocrystals (4–25 nm range) with an average size of ca. 12 nm. As should be expected, it was difficult to observe the HESOx particles when confined in the OLC because of the high carbon content of the crystalline OLC (∼90%), as shown by the TGA data (Figure S4). The lattice fringe spacings of the HESOx (Figure S5) were measured as 0.25, 0.29, and 0.47 nm, which correspond to the interplanar spacing of (311), (220), and (111) planes of a spinel crystalline structure, respectively. For HESOx/OLC (Figure S6), an interlayer spacing of 0.337 nm was observed, which corresponds to the graphene (002) plane. TGA results show that thermal stability decreases as HESOx≫ OLC > HESOx/OLC > HESOx/OLC_AT_, confirming the successful integration of the HESOx with OLC and the subsequent conversion of the pristine HESOx/OLC to the HESOx/OLC_AT_.

Figure 1A shows a scanning transmission electron microscope (STEM) high-angle annular dark-field (HAADF) image (Figure 1B) of the HESOx/OLC_AT_ showing the image along the [111] axis of the inverse spinel. Except for Cu, all the other elements (Fe, Ni, Co, and Ni) are relatively uniformly distributed in the solid solution. The inability of Cu to fully become part of the solid solution could be due to Jahn–Teller effect, whereby the oxygen sublattice around the Cu ion is locally deformed, making it difficult for CuO to diffuse into the spinel phase.^11^

**Figure 1:**
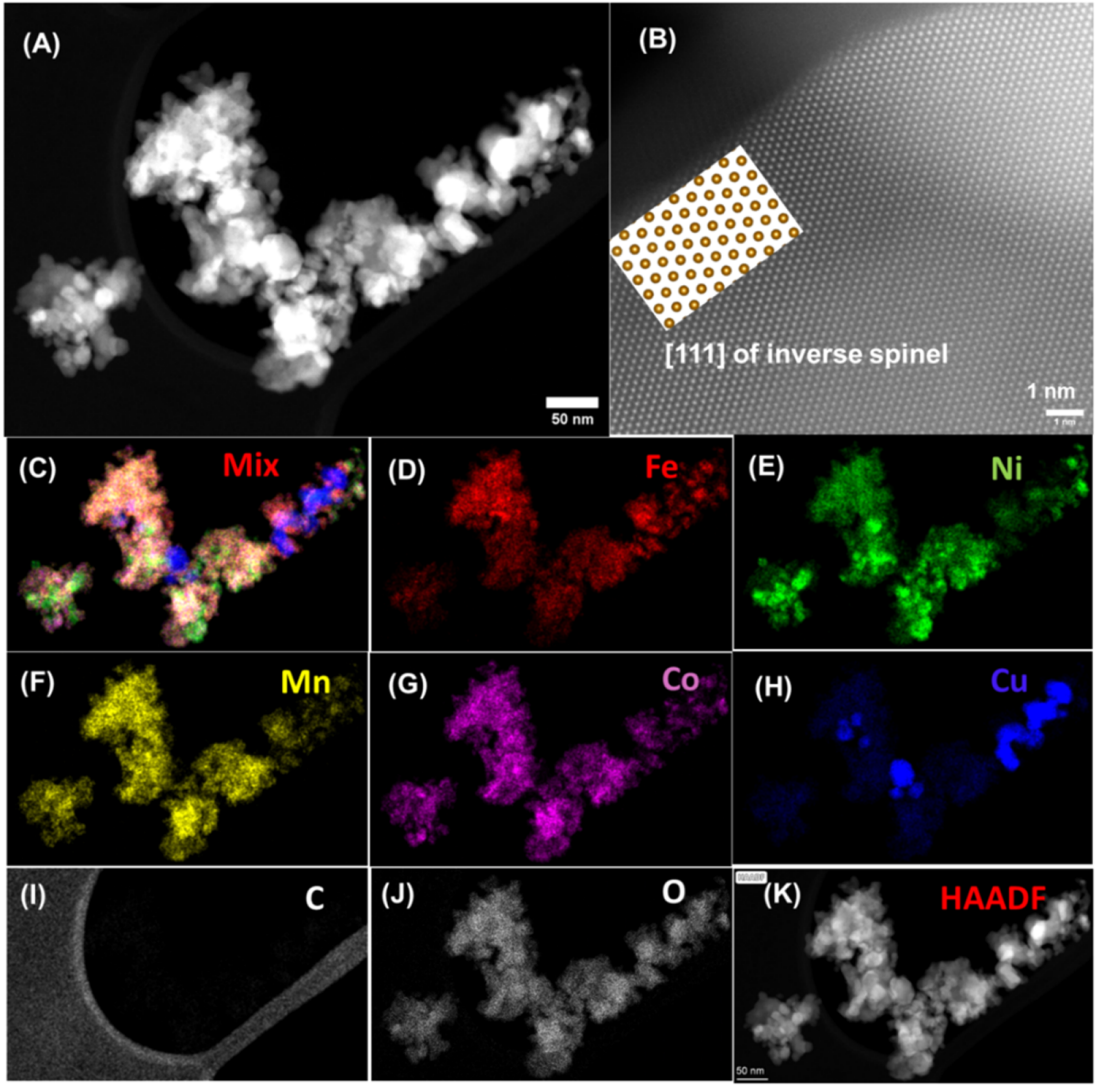
STEM HAADF images of a typical HESOx/OLC_AT_: (A) Particle STEM image of HESOx/OLC_AT_; (B) STEM-HAADF image of HESOx/OLC_AT_ particle, (C–J) STEM EDX maps of individual elements and (K) HAADF of the particle.

The schematic crystal structure of the HESOx material is shown in Figure 2A, while Figure 2B represents the powder X-ray diffraction patterns of HESOx studied in this work. The diffraction peaks appear at 18.4, 30.4, 35.7, 37.4, 43.5, 53.9, 57.6, and 62.5° correspond to the (111), (220), (311), (222), (400), (422), (511), and (440) Miller indices, respectively. These diffraction peaks are typical of the conventional inverse spinel oxides. The dominance of the (311) peak in the XRD pattern is a clear indication that these materials assume the crystal structure of an inverse spinel such as Fe_3_O_4_ or NiFe_2_O_4_. The distinct peak at 38.8° is related to CuO. It is suspected that the Jahn–Teller effect could have led to the isolation of Cu, whereby the oxygen sublattice around the Cu ion is locally deformed, making it difficult for CuO to diffuse into the spinel phase.^11^ Since there is no existing crystallographic information file (.cif) for the HESOx in the literature, we obtained one by using naturally occurring Fe_3_O_4_ as the parent inverse spinel structure. As seen in Figure 2B, the Bragg lines essentially matched the conventional Fe_3_O_4_ inverse spinel structure; the slight differences should be expected as HESOx (unlike the Fe_3_O_4_ that contains just one metal) contains five different transition metals with different atomic radii that can lead to distortion in the crystal lattice. Zooming in on the (311) plane (Figure 2C), one can observe a downward shift of 2θ degrees of 35.72, 35.31, and 35.00° for HESOx, HESOx/OLC, and HESOx/OLC_AT_, respectively, indicating the largest lattice expansion for HESOx/OLC_AT_. Similarly, the carbon plane (002) showed the highest lattice expansion for HESOx/OLC_AT_ (2θ = 24.24 °) compared to 25.60 ° for both HESOx and HESOx/OLC. The result suggests that the mild acid treatment increases the lattice of HESOx/OLC, which is advantageous for improved ionic transport.

**Figure 2:**
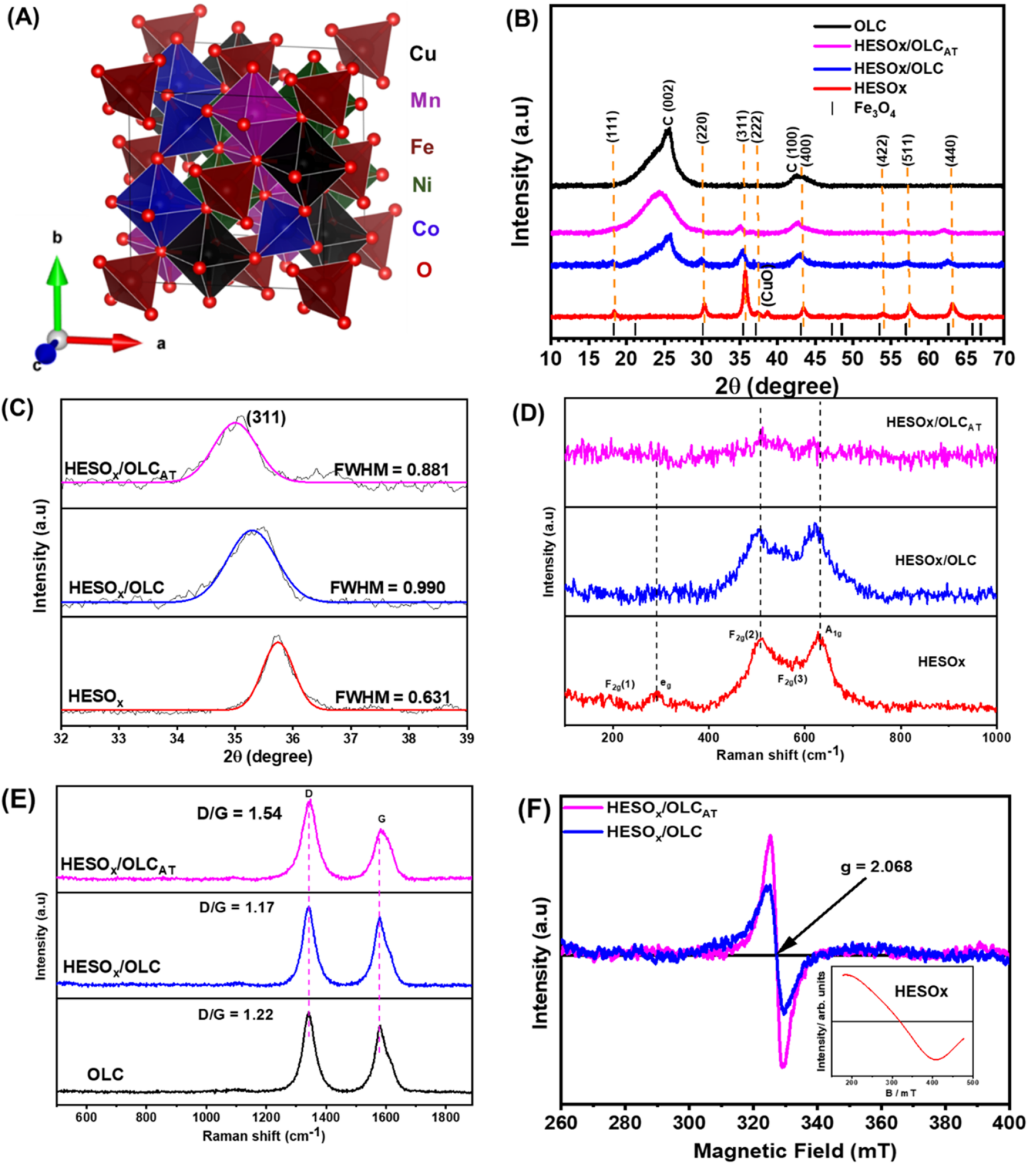
(A) Schematic crystal structure of the HESOx material studied in this work uses Fe_3_O_4_ as a parent spinel structure. The powder XRD patterns of (B) HESOx, HESOx/OLC, HESOx/OLC_AT_, and OLC modeled with the Bragg lines of the parent Fe_3_O_4_ inverse spinel oxide. (C) Zoomed-in (311) plane for HESOx, HESOx/OLC, and HESOx/OLC_AT_. Raman spectra taken at the expected regions for the (D) spinel oxide and (E) carbon. (F) EPR spectra of HESOx/OLC and HESOx/OLC_AT_, with the inset showing the EPR of the pristine HESOx.

Figure 2D compares the Raman spectra of the various HESOx materials in the band regions between 100 and 1000 cm^–1^. The bands around 200, 300, 500, 600, and 700 cm^–1^ are characteristic Raman active modes of spinel metal oxides and are related to F_2g_, E_g_, F_2g_, E_2g_, and A_1g_, respectively. The symmetric stretching mode (A_1g_) is characteristic of the spinel oxide structure (M–O), which is due to the vibration of the octahedral M–O bond. F_2g_ is the symmetric bending mode associated with the vibration of the tetrahedral sites, while E_g_ is the symmetric deformation mode due to the octahedral sites.

The Raman bands, notably A_1g_, shifted to a lower region, i.e., from HESOx (631 cm^–1^) to HESOx/OLC (624 cm^–1^) and HESOx/OLC_AT_ (614 cm^–1^). The downward shift is indicative of an increased concentration of oxygen defects, which has been linked to enhanced catalytic activity.^12^ Of particular interest is that HESOx/OLC_AT_ shows the weakest intensity of bands, which may be attributed to increased defects. The increase in oxygen defects may be related to the higher surface area of HESOx/OLC_AT_ particles stabilized in nanopockets within the OLC matrix. In other words, HESOx/OLC_AT_ is more defective than its HESOx/OLC and HESOx counterparts. The G band relates to the sp2 hybridized carbon atoms (i.e., graphitic structure) while the D band is due to the high concentration of sp3 hybridized carbon atoms (i.e., defective graphitic structure). The ratio of the area under the D and G bands (i.e., *I*_D_/*I*_G_, Figure 2E) decreased as HESOx/OLC_AT_ (1.54) > OLC (1.22) > HESOx/OLC (1.17), meaning that HESOx/OLC_AT_ is more defective than OLC and HESOx/OLC. The increased sp^3^ hybridized carbon is an indication of strong metal oxide-support interactions, such as covalent bonds between the metal oxide and support, improving stability and charge transfer between the HESOx and carbon.^13^ Electron paramagnetic resonance (EPR) spectroscopy provides a direct measure of the concentration of the paramagnetic species in the sample. O^–^ is of particular interest as an indicator of the surface defect concentration, generally grouped as oxygen vacancies in the literature.^14,15^ Figure 2F shows the typical room-temperature EPR spectra of HESOx/OLC and HESOx/OLC_AT_. EPR was used to further interrogate the presence of oxygen vacancies. While the OLC-based HESOx composites showed strong EPR signals (Figure 2F), the pristine materials did not show any useful signal (not shown here), indicating that OLC-based HESOx composites possess high concentrations of unpaired electron contents, arising from the electronic interactions between the OLC and HESOx materials. The EPR signals at *g* = 2.068 are associated with the electron capture on the oxygen vacancies, with HESOx/OLC_AT_ exhibiting a stronger peak intensity (i.e., more oxygen vacancies) than HESOx/OLC. The EPR finding corroborates the Raman data. It is well established that defect engineering (oxygen vacancies) provides a unique opportunity to modulate the electronic properties of metal oxides for improved electrocatalytic activities. As would be shown later, HESOx/OLC_AT_ with the highest content of oxygen vacancies gives the best electrocatalytic activities for RZAB. This should perhaps not be surprising, since it is known that oxygen vacancies increase in amorphous oxide materials,^16,17^ and boost electrocatalytic response because of the absence of well-defined crystal planes.

Figure 3 depicts the high-resolution XPS survey scans of the pristine HESOx and its products upon subjection to EG-water treatment to give HESOx/OLC and acid–base treatment for defect-rich HESOx/OLC_AT_. The full XPS spectra confirm the presence of elements Cu, Mn, Fe, Ni, Co, and O, in agreement with the EDS results. It is interesting to observe that the XPS survey scan (repeated in both laboratories in the US and SA) shows excellent consistency, whereby surface OLC dominates the HESOx (Figure 3A), while mild acid treatment leads to defective HESOx protruding out of the high-curvature and defective OLC surface (Figure 3C). This protrusion may be related to a type of “tip effect”, which is known to enhance electrocatalysis by creating in situ strong local electric field around the catalytic metal site.^13,18^

**Figure 3:**
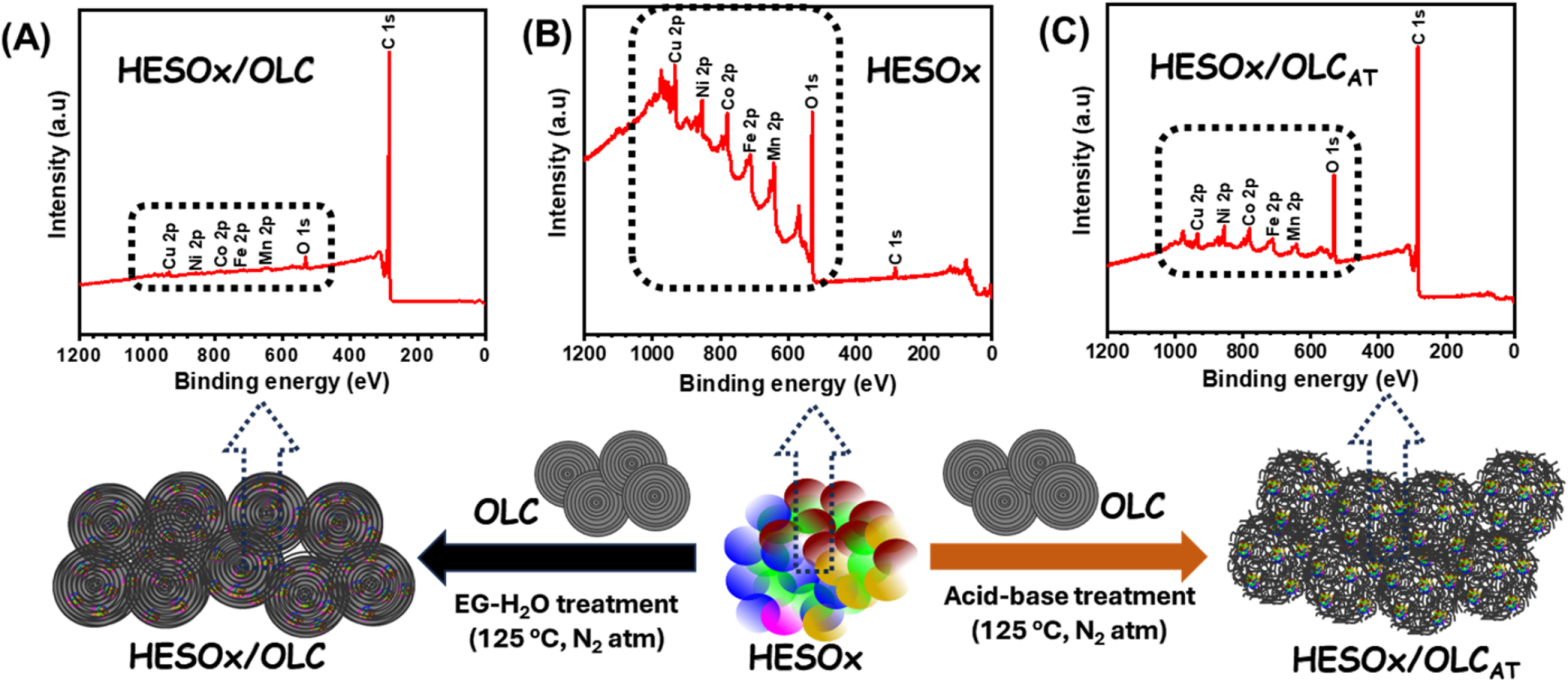
High-resolution XPS survey scan for (A) HESOx/OLC, (B) HESOx, and (C) HESOx/OLC_AT_ with a corresponding carton depiction of the two key products (HESOx and HESOx/OLC_AT_) from their synthesis protocols. Note the protrusion of the surface of HESOx out of the defective OLC, confirmed by the XPS survey scan.

Next, the XPS spectra of each element in the HESOx were studied to understand the chemical states and structures of the prepared HESOx samples. All of the spectra were successfully fitted using Gaussian–Lorentzian functions after the Shirley background subtraction. Figure 4 represents the deconvoluted high-resolution spectra of Mn 2p (Figure 4A), Fe 2p (Figure 4B), Ni 2p (Figure 4C), Co 2p (Figure 4D), Cu 2p (Figure 4E), and O 1s (Figure 4F) in the pristine and OLC-based HESOx complexes. Each of the M 2p was deconvoluted into two spin–orbit doublets combined with two visible shakeup satellite peaks (abbreviated as “sat” in the figure). For each of the metals (Mn 2p (∼641.9 eV), Fe 2p (∼711.8 eV), Ni 2p (∼854.3 eV), Co 2p (∼779.8 eV), and Cu 2p (∼933 eV)), the HESOx/OLC_AT_ exhibited the most positive shifts of the deconvoluted M^2+^ and M^3+^ peaks compared to the HESOx/OLC and pristine HESOx complexes, which confirms electron transfer from the metallic elements to the highly curved and defective OLC surface. Importantly, such positive shifts indicate weak d-band centers (especially for the HESOx/OLC_AT_ catalyst) that bode well for enhanced ORR and OER catalysis.^19^ For Cu 2p (∼ 933 eV), the pristine HESOx exhibited metallic Cu^o^ (930.371 eV) in addition to Cu^+^ (932.991 eV) and Cu^2+^ (934.813 eV). The OLC-based HESOx complexes only showed the Cu^+^ and Cu^2+^, with very minor positive shifts (∼0.2 eV), which indicates that Cu does not significantly contribute to the electron transfer processes in the HESOx complexes.

**Figure 4:**
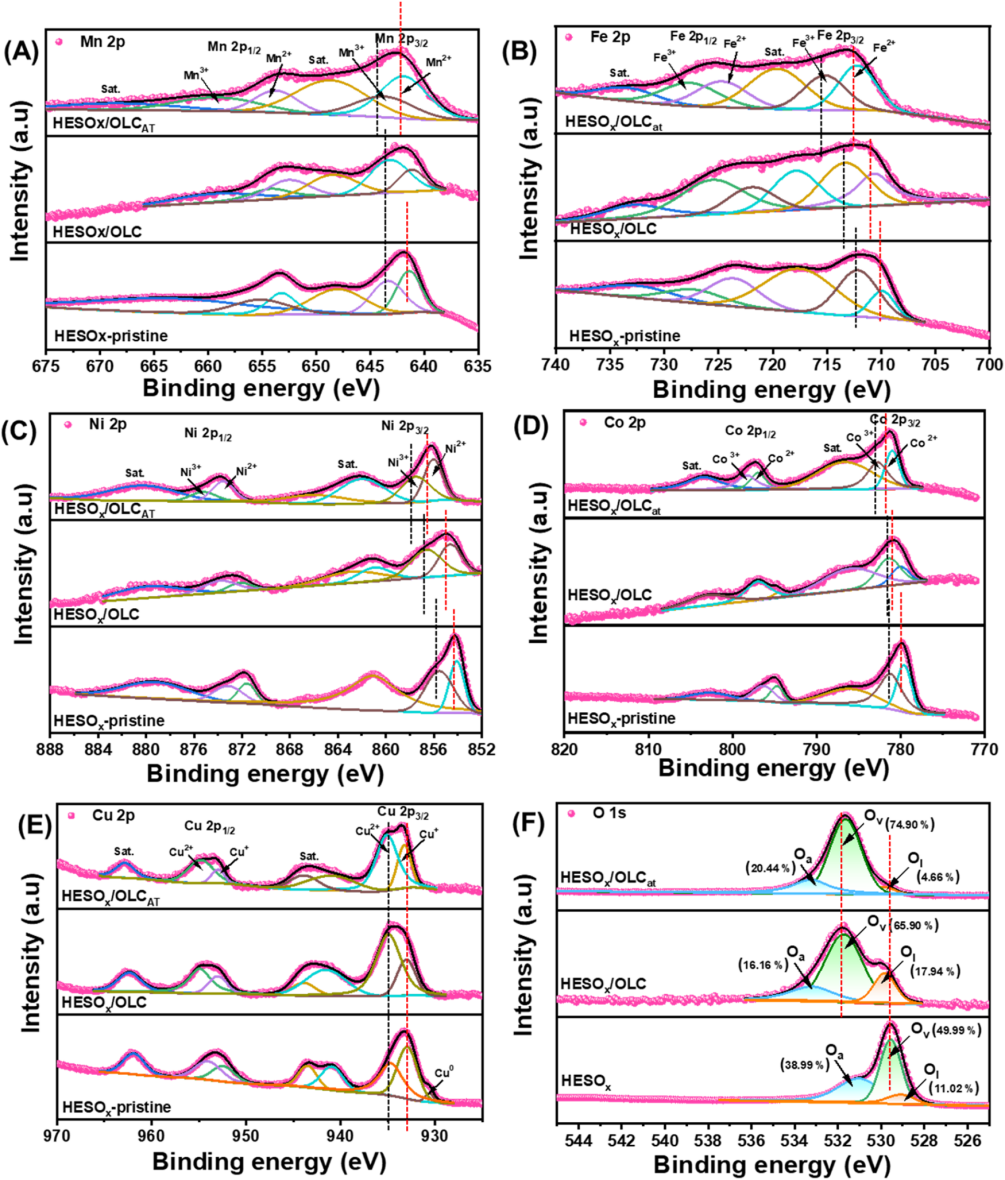
XPS spectra of (A) Mn 2p, (B) Fe 2p, (C) Ni 2p, (D) Co 2p, (E) Cu 2p, and (F) O 1s of the pristine HESOx, HESOx/OLC, and HESOx/OLC_AT_.

The O 1s deconvoluted XPS is shown in Figure 4F. Upon introduction of the OLC to the HESOx, the peak shifted to a higher binding energy: pristine HESOx (529.6 eV) < HESOx/OLC ≈ HESOx/OLC_AT_ (531.7 eV). This oxidative shift further confirms the electronic interaction between the OLC and HESOx species. Such electronic modulation of the HESOx by the highly curved OLC should lead to improved electrocatalysis.^20,21^ Each of the spectra was deconvoluted into three peaks with the pristine HESOx at 529, 529.5, and 531.2 eV, which are associated with the surface lattice oxygen (O^2–^) bound to metals (i.e., metal–oxygen bonds) (O_lat_), defect sites/surface oxygen species (O_2_^2–^/O^–^) from low coordinated oxygen atoms or vacancies (O_vac_), and surface-adsorbed oxygen species such as water molecules (O_ads_), respectively.^14,15^ For both HESOx/OLC and HESOx/OLC_AT_, the peaks of O_lat_, O_vac_, and O_ads_ appear at the same peaks of 529.8, 531.7, and 533.2 eV, respectively. The ratio of O_vac_/O_lat_ (peak area) increases as HESOx/OLC (3.67) < HESOx (4.54) < HESOx/OLC_AT_ (16.07), indicating a higher concentration of surface oxygen vacancies (defects) on the HESOx/OLC_AT_. It has been well established that oxygen vacancies play vital roles in promoting electrocatalytic oxygen reactions (ORR and OER). In the presence of vacant oxygen sites, oxygen species can be easily adsorbed and desorbed, thus permitting more efficient mass transport processes to be realized. The deconvoluted C 1s spectra of the three materials (Figure S7) show that the HESOx/OLC_AT_ carbon becomes less graphitic (42.46% sp^2^) than the HESOx/OLC (52.60% sp^2^). The XPS finding on the existence of oxygen vacancies strongly corroborates the results of both the EPR and Raman experiments.

The nitrogen adsorption–desorption curves of the HESOx samples (Figure S8) depict a characteristic type IV isotherm. From the Brunauer–Emmett–Teller (BET) data (Table S1), HESOx/OLC_AT_ gave the largest BET specific surface area (SBET) of 434.21 m^2^ g^–1^, compared to 387.97 and 14.83 m^2^ g^–1^ for HESOx/OLC and pristine HESOx, respectively. From the BET data, HESOx/OLC_AT_, with its high SBET and large pore volume, will have more exposed catalytically active sites for enhanced OER, ORR, and RZAB activities.

### Half-Cell Electrochemical Evaluation

The 3-electrode electrochemical experiments were carried out in 1 M KOH, but only for HESOx/OLC and HESOx/OLC_AT_, as the pristine HESOx was insoluble in the ink solvents used for electrode modification. As shown in Figure 5A, the CVs of the HESOx/OLC and HESOx/OLC_AT_ obtained in nitrogen-saturated 1 M KOH solution gave broad redox peaks of similar half-wave potential (*E*_1/2_ ∼ 0.73 V vs RHE) due to the M­(II)/M­(III) redox process (where M is most likely due to Mn, Co, and Fe, as Cu is redox-inactive, while Ni redox usually appears at the higher-potential region). The broadness of the redox peak is indicative of 2 or more metals involved in the process. The peak-to-peak separation potential (Δ*E*_p_/V) of HESOx/OLC (0.33 V) is larger than that of HESOx/OLC_AT_ (0.22 V), meaning that HESOx/OLC_AT_ exhibits faster electron transfer kinetics than HESOx/OLC. The electrochemical active surface area (ECSA) was determined from the capacitive region of the CV evolutions (Figure S9), calculated as ca. 331 and 405 cm^2^ for HESOx/OLC and HESOx/OLCAT, respectively. This trend is consistent with the BET data, corroborating the higher surface area of HESOx/OLC_AT_ over that of its HESOx/OLC counterpart.

**Figure 5:**
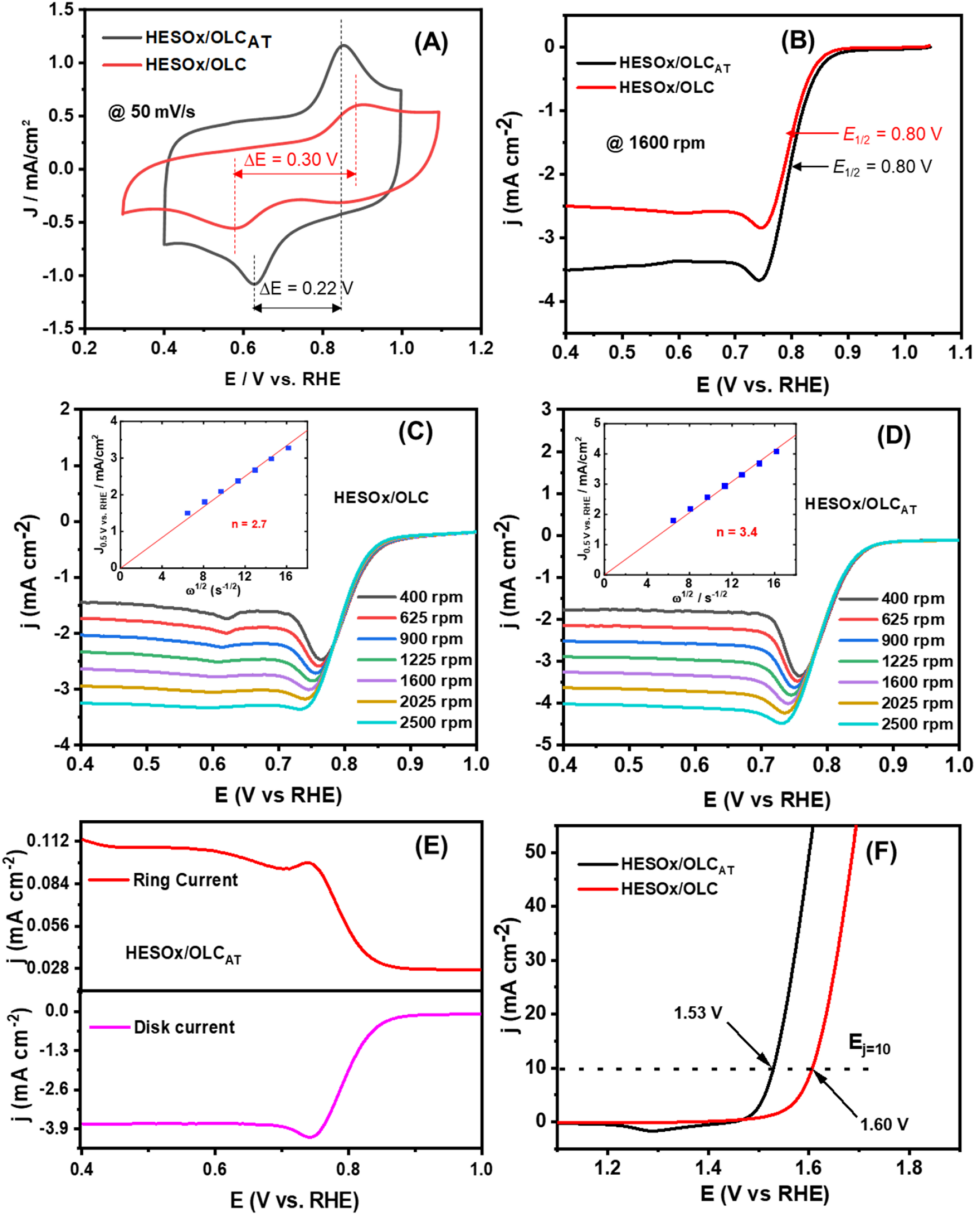
(A) Comparison of the redox processes of HESOx/OLC and HESOx/OLCAT at 50 mV/s in nitrogen-saturated 1 M KOH solution. (B) ORR polarization curves at 1600 rpm in oxygen-saturated 1 M KOH solution. Comparison of the ORR polarization curves for (C) HESOx/OLC and (D) HESOx/OLCAT at different rotation speeds (400–2500 rpm). The insets in (C, D) represent the Koutecky–Levich plots of HESOx/OLC and HESOx/OLCAT, respectively. (E) RRDE voltammograms of HESOx/OLC_AT_ catalysts for the ORR in O_2_-saturated 1 M KOH. Ring electrode currents and disk electrode currents are normalized to the geometric surface area of the disk electrode. (F) OER polarization curves at 1600 rpm in O_2_-saturated 1 M KOH. Scan rate: 5 mV s^–1^; rotation rate: 1600 rpm. The catalyst loading was 24 μg_metal_ cm^–2^, and the ring potential was 1.2 V.

In oxygen-saturated KOH electrolyte solution, both catalysts showed well-defined diffusion-limited current densities (*j*_lim_) of ca. 0.25 and 3.5 mA cm^–2^ at 1600 r.p.m for HESOx/OLC and HESOx/OLC_AT_, respectively, and similar *E*_1/2_ values of 0.80 V (Figure 5B). The corresponding Koutecky–Levich (K–L) plots (*j* ^–1^ vs ω^–1/2^, insets of Figure 5C,D) obtained from the RDE voltammograms at 0.75 V are characterized by excellent linearity (*R* ^2^ ≈ 0.99), meaning first-order reaction kinetics for the ORR as a function of the concentration of dissolved oxygen. The number of transferred electrons at +0.75 V was calculated to be ∼2.7 for HESOx/OLC and 3.4 for HESOx/OLC_AT_, confirming the higher ORR activity of HESOx/OLC_AT_ than that of HESOx/OLC. The HESOx/OLC_AT_ catalysts gave better ORR kinetics (Tafel slope of ca. 51 mV dec^–1^) than HESOx/OLC (Tafel slope of ca. 70 mV dec^–1^) (Figure S10B). However, both electrocatalysts displayed lower than the 4-electron transport for complete conversion of oxygen to water due to the possibility of H_2_O_2_ generation. Evaluation of H_2_O_2_ generation is critical, as H_2_O_2_ has the potential to deteriorate the performance of fuel cells or metal-air battery membranes. Figure 5E exemplifies typical RRDE voltammograms of the HESOx/OLCAT catalyst in an O_2_-saturated 1 M KOH solution. Both ring and disk currents are normalized to the geometric surface area of the disk electrode. The ring current is much smaller (0.10 mA cm^–2^) than the disk current (∼3.9 mA cm^–2^) at ca. 0.75 V and reached a maximum at 0.4 V. The plots of the calculated electron transfer number and the H_2_O_2_ yield vs potential are shown in Figure S11. The calculated electron transfer number is ∼3.0 at 0.75 V, and the H_2_O_2_ yield was about 50%. Figure 5F compares the OER polarization curves of HESOx/OLC and HESOx/OLC_AT_, with overpotentials of about 370 and 300 mV at 10 mA cm^–2^, respectively. The HESOx/OLC_AT_ catalysts gave better OER kinetics (Tafel slope of ca. 48 mV dec^–1^) than HESOx/OLC (Tafel slope of ca. 83 mV dec^–1^) (Figure S10C).

For application in metal-air batteries such as ReZAB, it is important to determine the “bifunctionality indices (BI)” of these catalysts. Bifunctionality index is a measure of a catalyst’s ability to allow both the forward (OER) and reverse (ORR) reactions of the air cathode to occur. The smaller the BI value, the faster the kinetics of the reaction and vice versa. From the results (Figure S10A), it was found that the change in potential (Δ*E*/V) between ORR and OER follows this sequence: HESOx/OLC_AT_ (Δ*E* = 0.70 V) < Pt/C-IrO_2_ (Δ*E* = 0.75 V) < HESOx/OLC (Δ*E* = 0.78 V), meaning that the HESOx/OLC_AT_ gave the best electrocatalytic kinetics for application in ReZAB. Indeed, the low-voltage hysteresis of the HESOx/OLC_AT_ is noteworthy^22^ and even much lower than generally seen in the current literature that shows >1.0 V.^23^

### Theoretical (DFT) Calculations

Although DFT calculation was not the main focus of this work, it was deployed to simply provide some insights into three expected phenomena, i.e., energy band structure, d-band centers, and the d-π hybridization. DFT calculations were carried out using O_2_, *O, *OH, and *OOH as adsorbates for ORR and OER. Figure 6 exemplifies the electronic spectroscopic data of the adsorbates on defective HESOx/OLC (i.e., HESOx_d_/OLC_d_, representing the HESOx/OLC_AT_) showing the adsorption of O* (Figure 6A) and formation of *OH (Figure 6B) which are generally associated with the intermediates for the rate-determining step for ORR^24,25^ and OER,^26,27^ respectively. The two prominent bands observed are the d- and p-bands, with the latter dominating due to the high concentration of OLC. The same trend was observed for the other intermediates O2 and *OOH (not shown). The energy band structure of the HESOx/OLC_AT_ is wide, dense, and uniform, which means uniform and well-distributed electronic states.^28^ Such a uniform density of states provides reactive sites in catalytic reactions. The wide distribution of electrons means that the electronic interactions between the surface of the HESOx/OLC_AT_ and the reacting intermediates will be diverse, which will bode well for enhanced reactivity of the ORR/OER and catalyst’s stability. Such a dense energy band structure indicates the high tunability and responsiveness of the electrons in HESOx/OLC_AT_, which is critical for enhanced electrocatalysis, including fast electron transfer kinetics.

**Figure 6:**
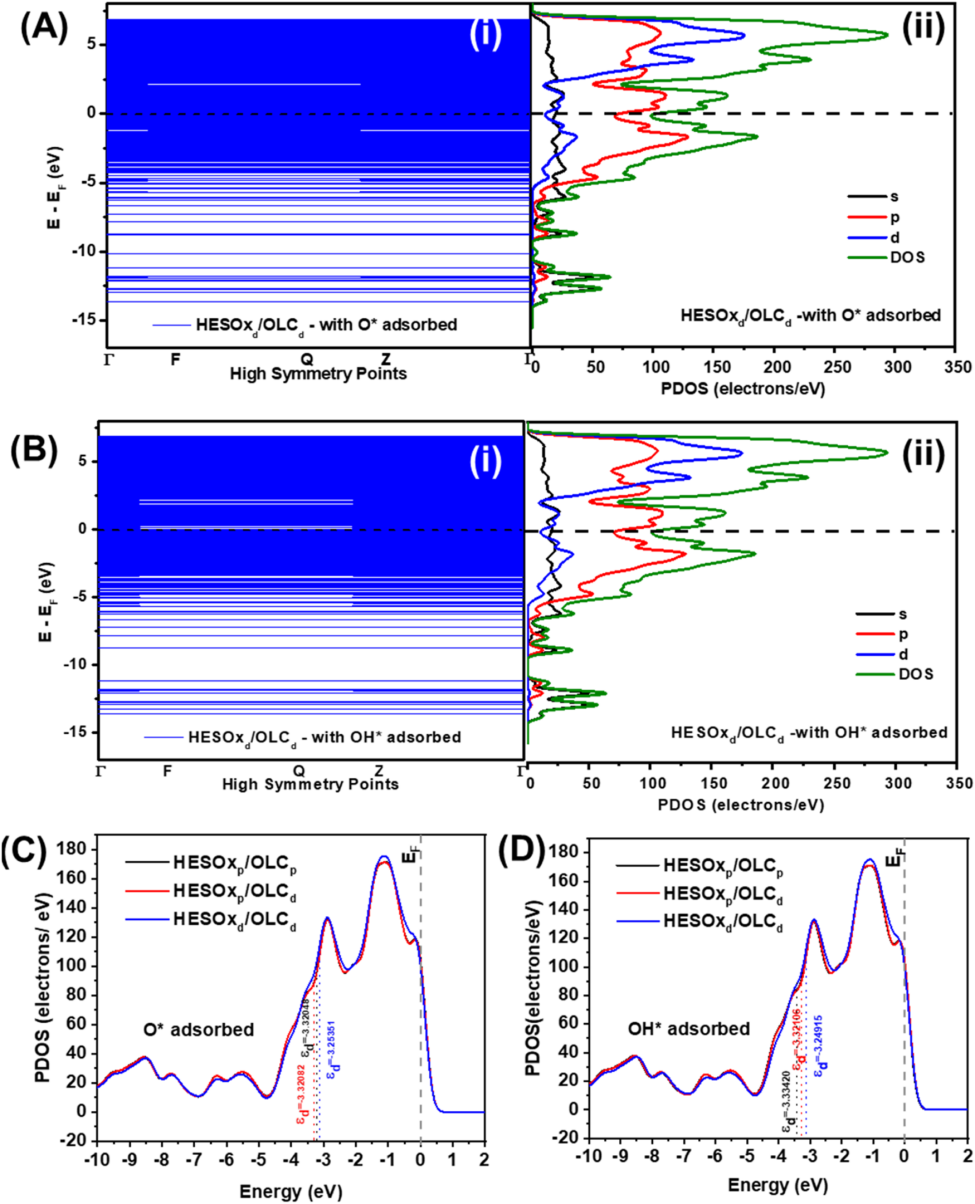
Typical electronic spectroscopic data of the defective HESOx_d_/OLC_d_ (i.e., equivalent of HESOx/OLC_AT_) for the ORR/OER intermediates: (A) O* and (B) OH*. Note that (i) and (ii) represent the band structure and PDOS, respectively. The d-band centers calculated for (C) O* and (D) OH* calculated for the HESOx_p_/OLC_p_, HESOx_p_/OLC_d_, and HESOx_d_/OLC_d_.

The location of the d-band center is the result of the d-π atomic orbital hybridization. The d-band center (ε_d_/eV) is one possible determinant of the electrocatalytic activity of transition metal catalysts. The d-band center (ε_d_) was calculated according to eq 7 ^29,30^

7 $$\varepsilon_{d}=\frac{\int \rho EdE}{\int \rho dE}$$

where ε_d_ is the d-band center, ρ is the density of the d-band, *E* is the energy of the d-band, and ρd*E* represents the number of states.

Figure 6C,D compares the respective d-band centers of the *O and *OH calculated for the HESOx_p_/OLC_p_, HESOx_p_/OLC_d_, and HESOx_d_/OLC_d_. To improve the ORR and OER activities, the d-band center of surface HESOx must be downshifted, which, in principle, can be realized if the transition metals in HESOx are further oxidized as already shown via the XPS data. As shown in the Supporting Information (Table S2), the d-band centers for HESOx are much stronger (around −1.9 eV) than the OLC-based HESOx (around −3.3 eV) for each of the intermediates. Interestingly, however, the d-band centers recorded at the HESOx_d_/OLC_d_ surface for *O (−3.25351 eV) and *OH (−3.24195 eV) are slightly more positive (i.e., slightly stronger adsorption, being closer to the Fermi level) than the other electrocatalysts studied in this work. The reason for this slight deviation from the d-band theory has been observed by other workers and was attributed to the ability of the adsorbate to adsorb at different sites on the catalyst.^29,31,32^ Thus, it is highly probable that this could be happening in our case, too, especially considering the inherent ability of high-entropy electrocatalysts to provide multiple catalytic sites for intermediates coupled with the highly defective nature of the HESOx/OLC_AT_ catalyst studied in this work.

### Accelerated Degradation Test (ADT)

Based on the high electrocatalytic performance of HESOx/OLC_AT_ toward the ORR and the OER processes, all further studies were focused on the HESOx/OLC_AT_ catalyst. First, we investigated its ORR electrochemical durability via an accelerated degradation test (ADT). As shown in Figure 7A, the initial CV showed two broad redox peaks that disappeared after 10,000 cycles. From the 10,000th cycle, a rectangular CV shape, characteristic of capacitive behavior, was observed until the 30,000th cycle, demonstrating a remarkable enhancement in stability.

**Figure 7:**
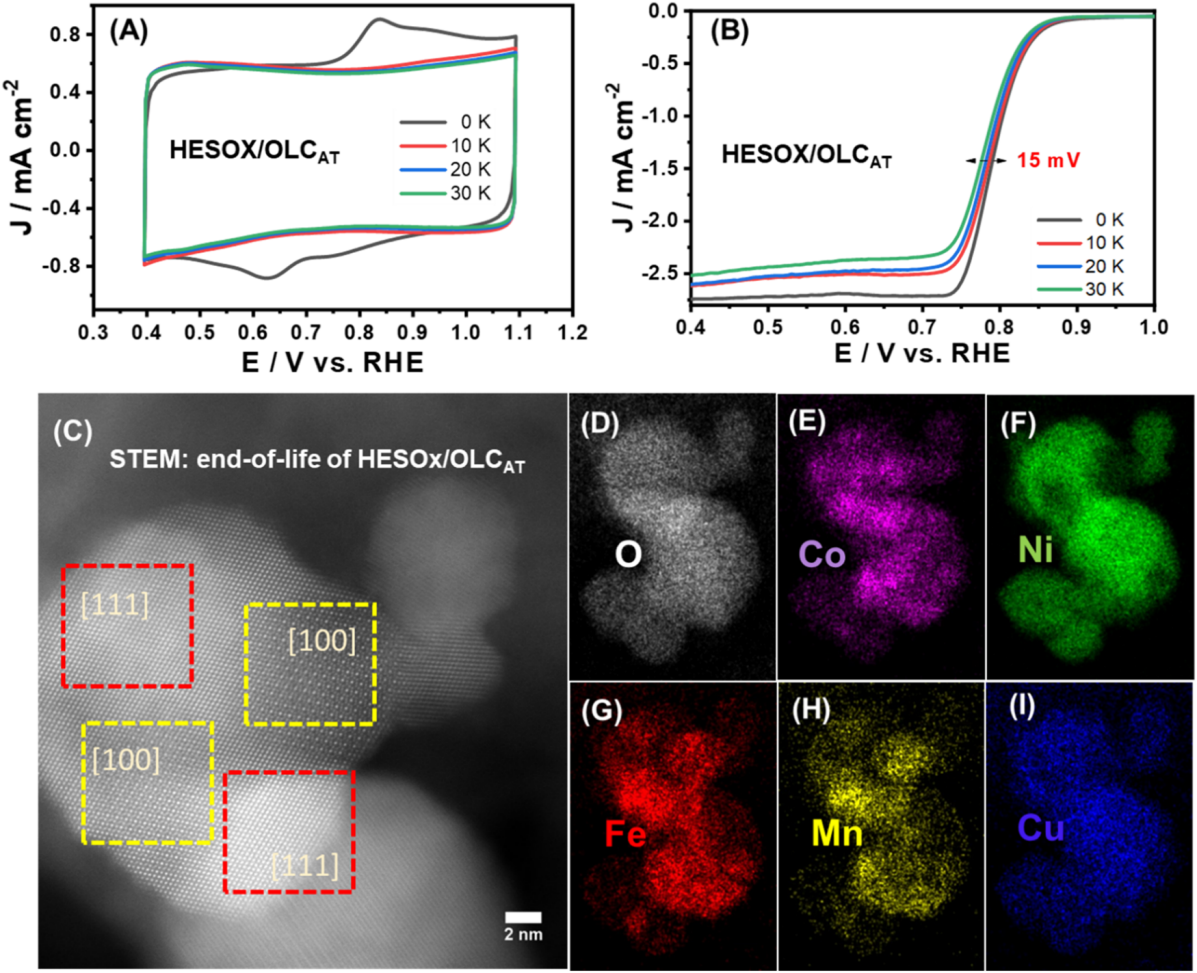
(A) CV and (B) RDE evolutions (1600 rpm) of the HESOx/OLC_AT_ catalyst before and after ADTs (10−30,000 cycles) in oxygen-saturated 1 M KOH at 0.85 V vs RHE. (C) Postmortem analysis: HAADF image at the end-of-life HESOx/OLC_AT_ after ADTs (30,000 cycles) showing no noticeable structural change from its original inverse spinel structure, and (D-I) individual STEM-EDX map of the various elemental composition of the HESOx/OLC_AT_.

As exhibited in Figure 7B, the HESOx/OLC_AT_ catalyst showed an activity decay with a Δ*E*_1/2_ value of 15 mV after 30,000 cycles. To interrogate the reason for the enhanced electrocatalytic activity, HAADF-STEM characterization (Figures 7C and S12) was further carried out to examine the size, morphology, and crystal phase changes. Impressively, after the ADT of 30,000 cycles, the HESOx/OLC_AT_ catalyst retained its pristine inverse spinel structural properties, with EDX mappings (Figure 7D–I) showing uniform elemental distribution. Furthermore, using chronoamperometry to test the OER stability (Figure S13), it was shown that the HESOx/OLC_AT_ maintained a constant voltage of 1.53 V for the 10 h duration, while the HESO_*x*_/OLC showed a higher voltage (1.66 V) and quickly degraded after 6 h. This result proves that the HESO_*x*_/OLC_AT_ catalyst is more electrochemically robust than the HESO_*x*_/OLC.

### Evaluation of Rechargeable Zinc–Air Battery for Technological Application

Figure S14 exemplifies typical discharge polarization curves of HESOx/OLC and HESOx/OLC_AT_ with satisfactory power densities of 139 and 152 mW cm^–2^, respectively (Figure S14A). Figure S14B shows typical discharge–charge polarization curves of the HESOx/OLC_AT_ for the RZAB at a constant current density of 2 mA cm^–2^ carried out for up to 300 cycles. Also, discharge–charge experiments were conducted for the ReZAB cell at 2 mA cm^–2^ and low cycling time of 1 h per cycle (i.e., shallow cycling), which exhibited long-hour cycling (>300 h) and good kinetics (Δ*E* = 58–74 mV) (Figure S15). Shallow cycling for ReZAB cells, as shown in Figure S15, provides no useful data for real application. Disappointingly, however, shallow cycling (as low as 10 min per cycle) is what is mostly reported in the literature to prove “high-performance” for both conventional electrocatalysts^33–36^ and high-entropy electrocatalysts^28,32,37–40^ (also see Table S3 and references therein). It cannot be overemphasized that shallow cycling alone should not be used to prove excellence in performance. This is why, for technological relevance, Rolison and co-workers^2^ recommended the need for ReZAB to be subjected to deep cycling at a current loading of 10 mA cm^–2^ and at 35 mWh cm^–2^ to be able to compete with Li-ion battery pack.^2^

In this work, the HESOx-based ReZAB was fabricated with a polished zinc plate as the anode, HESOx material as the cathode, and an alkaline aqueous electrolyte (6 M KOH + 0.2 M zinc acetate). For comparison, a ReZAB with commercial Pt–C/IrO_2_ as the air cathode catalyst was also assembled. Figure 8 compares the electrochemical properties of the ReZAB studied in this work using HESOx/OLC_AT_ and commercial 10% Pt/C-IrO_2_ as air cathode electrocatalysts at 10 mA cm^–2^ current loading and different discharge areal energy densities of (A) 8 h per cycle and (B) 6 and 12 h per cycles. The double green arrow indicates the voltage difference between the charge and discharge cycles (Δ*E*/V).

**Figure 8:**
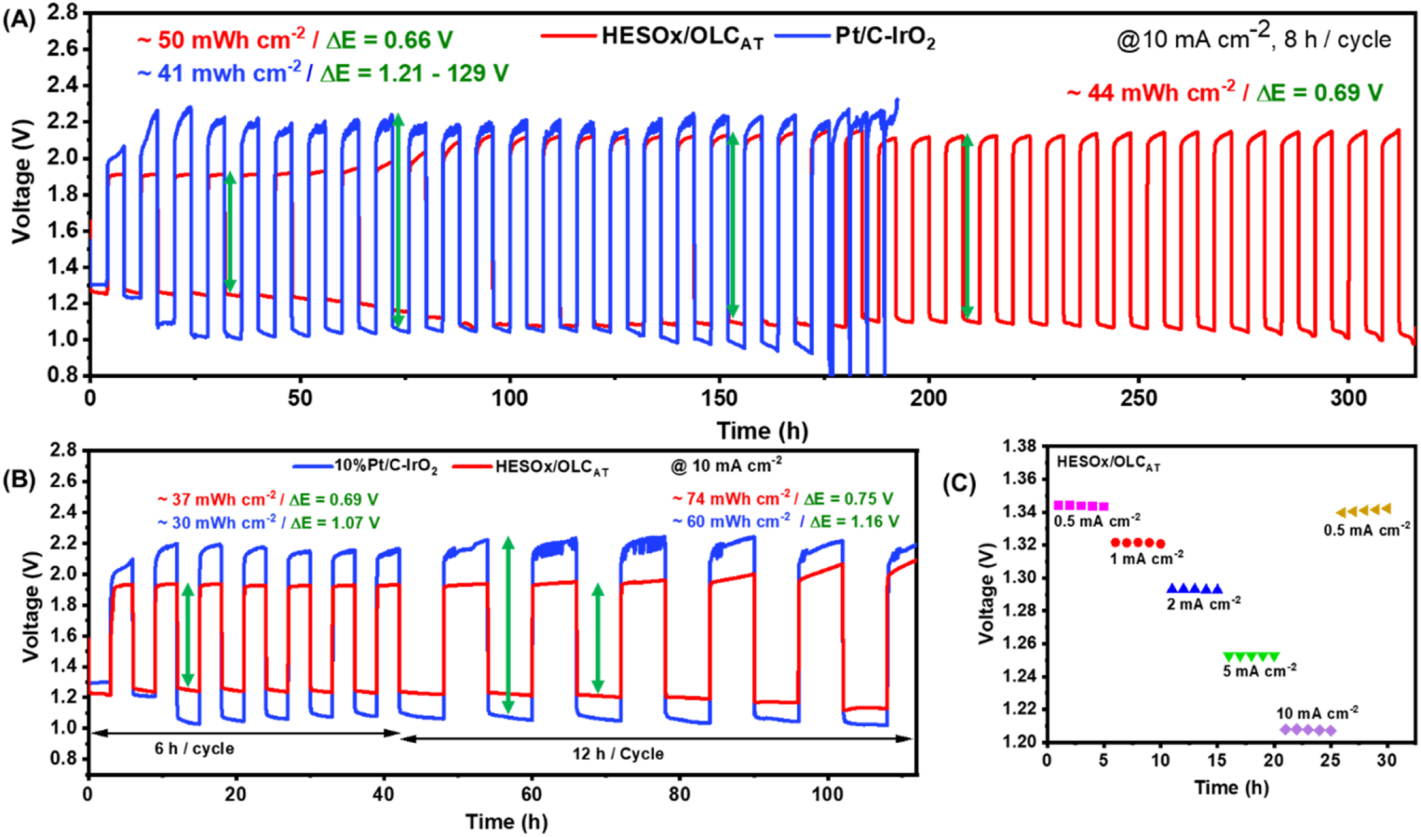
Electrochemical characterizations of HESOx/OLC_AT_ and commercial 10% Pt/C-IrO_2_ as air cathodes for aqueous rechargeable zinc–air batteries at 10 mA cm^–2^ current loadings and different discharge areal energy densities: (A) 8 h per cycle, (B) 6 and 12 h per cycles. (C) Rate capability of the ReZAB cell. The double green arrows in (A, B) depict the size of the voltage difference between the charge and discharge cycles (Δ*E*/V).

As seen from Figure 8A,B, the ReZAB can be discharged at higher areal energy density (37–74 mWh cm^–2^) than the recommended threshold of discharge areal energy density (35 mWh cm^–2^) and at excellent kinetics (Δ*E* = 0.66–75 V). In contrast, the highly expensive commercial dual-catalyst (Pt/C-IrO_2_) is unstable (crashes very easily) with poor kinetics (Δ*E* = 1.07–1.29 V). Figure 8C is the rate capability plot of the ReZAB cell, which reveals that the battery cell can be charged and discharged at different current densities. The results reported here for ReZAB compare with, and are even better, than the best 8% articles that were reported to have met the minimum threshold for real technological applications (please see Hopkins et al.^2^ for details). In addition, Table S3 compares the performance of HESOx/OLC_AT_ with some of the medium- to high-entropy material-based air-breathing catalysts used for ReZABs.^38,39,41–72^ It is clearly seen in Table S3 that most electrocatalysts use shallow discharge cycling (5–30 min) for ReZAB and expectedly achieved high number of cycles (as we also show in this work, see Figure S15), HESOx/OLC_AT_ achieved the rarely reported deep cycling (3–6 h) that would potentially allow for device applications. Indeed, the HESOx/OLCAT exhibits the most significant enhancement of a ReZAB yet reported.

## Conclusions

Single nanocrystals of inverse-type high-entropy spinel oxides confined in highly curved defective onion-like carbons (HESOx/OLC_AT_) have been successfully synthesized and characterized. The synthesis method adopted here results in controlled defective engineering (oxygen vacancies), strong electronic interaction between the OLC and HESOx, characterized by unique surface “tip” exposure of the metallic elements that allows for efficient electron transport from the metallic elements to the OLC species, and weak d-band centers of the component transition metals. The excellent electrocatalytic properties of defect-rich HESOx/OLC_AT_ toward ORR/OER and ReZAB are related to the strong electronic modulation arising from d-π hybridization and weakened d-band centers in the HESOx/OLC_AT_ complex. The level of electron transfer is more pronounced with Fe and less significant with Cu. It is shown that the ORR/OER activities in alkaline electrolyte are governed by the excellent electronic modulation of the defect-rich HESOx/OLC_AT_ catalyst with multiple catalytic sites for intermediates and weakened d-band centers of the rate-determining intermediates (i.e., *O adsorption for ORR and *OOH formation for the OER) compared to the pristine HESOx. The HESOx/OLC_AT_ showed excellent electrochemical cycling stability, proven by stable structural properties before and at end-of-life. The HESOx/OLC_AT_ catalyst was used to fabricate and test for application in ReZAB, and the battery was able to be continuously charged and discharged at a 10 mA cm^–2^ current load, yielding high areal energy densities between 37 and 74 mWh cm_geometric_^–2^. This result outperforms the literature-recommended threshold of 35 mWh cm_geometric_^–2^ for technological applications and represents one of the top performers for ReZAB reported. In the future, there will be a need to demonstrate this work using commercial-sized ReZAB cells.

## Supplementary Material



## Notes

Synthesis scheme for HESOx, HESOx/OLC, and HESOx/OLCAT; fabrication of the ReZAB cells; description of the computational studies; associated images and data for TGA, HRTEM, HRSEM, BET/Nitrogen adsorption/desorption curves; measurements of double-layer capacitance to determine the ECSA, ORR/OER bifunctional curves, and Tafel slopes for the catalysts; electron transfer number and amount of hydrogen peroxide (H_2_O_2_) produced in an alkaline medium for HESOx/OLCAT; STEM of the HESOx/OLCAT at the end-of-life HESOx/OLCAT after ADTs (30k cycles) showing no noticeable structural change from its original inverse spinel structure; long-term cycling tests, including chronoamperograms at 10 mA cm^–2^ for 10 h; discharge polarization curves and corresponding power density curves of the ReZAB based on the HESOx/OLC and HESOx/OLC_AT_ from LSV, typical discharge–charge polarization curves of the HESOx/OLC_AT_ based RZAB at a current density of 2 mA cm^–2^ between 5 and 300 cycles; discharge–charge curves of HESOx/OLC_AT_ and 10% Pt/C-IrO_2_ at a current density of 2 mA cm^–2^ for 1 h per cycle; tables showing Brunauer–Emmett–Teller (BET) data for the HESOx materials; summary of the d-band centers for ORR/OER intermediates; and survey of several recent literature (including this work) on rechargeable zinc–air batteries showing how this work outperforms most literature, especially in terms of areal-discharge energy density (PDF)
