# Supplementary material for: Defect-Engineered High-Entropy Spinel Oxide@Onion-Like Carbon Catalysts for High-Areal-Energy Rechargeable Zinc–Air Batteries
Source: Energy Fuels. 2025 Jun 25;39(27):13105–19. doi: 10.1021/acs.energyfuels.5c02012 (PMC12257454; doi:10.1021/acs.energyfuels.5c02012)
Supplement: Supplementary file 1 [file ef5c02012_si_001.pdf]

SUPPLEMENTARY INFORMATION

Defect-Engineered High-Entropy Spinel Oxide @ Carbon Onion Catalysts for High-Areal-Energy Rechargeable Zinc-Air Batteries

Agnes Mongwe[1], Aderemi B. Haruna[1], Lesego Gaolatlhe[1], Joesene Soto[2,3], Zixiao Shi[3,4], Patrick V. Mwonga,[1] Xiao-Yu Yang[1,5], David A. Muller[3,4], Héctor D. Abruña[2,*] and Kenneth I. Ozoemena[1,*]

[1.] *School of Chemistry, Molecular Sciences Institute, University of the Witwatersrand, Johannesburg 2050, South Africa.*

[2.] *Department of Chemistry and Chemical Biology, Cornell University, Ithaca, NY, United States.*

[3.] *Kavli Institute at Cornell for Nanoscale Science, Cornell University, Ithaca, NY, United States.*

[4.] *School of Applied and Engineering Physics, Cornell University, Ithaca, NY, United States.*

[5.] *State Key Laboratory of Advanced Technology for Materials Synthesis and Processing, School of Materials Science and Engineering, Wuhan University of Technology, Wuhan 430070, China*

[*]Authors to whom correspondence should be addressed: H.D. Abruña (e-mail: hda1@cornell.edu); K.I. Ozoemena (e-mail: Kenneth.ozoemena@wits.ac.za).

Electrochemical Measurements

The electrocatalytic active surface area (ECSA) of the HESOx/OLC and HESOx/OLC_{AT} catalysts was determined from CV experiments at low scan rates in the capacitive region (in this case, 0.18 – 0.38 V vs RHE). The measured charging current (i_c) is equal to the product of the scan rate (ν) and the electrochemical double-layer capacitance (C_{DL}), according to eqn. (1).

$$I_c = \nu C_{DL} \quad (1)$$

A plot of i_c with respect to ν yields a straight line with a slope equal to C_{DL} for the composite electrocatalysts. The ECSA is calculated from C_{DL} using eqn. (2):

$$\text{ECSA} = \frac{C_{DL}}{C_S} \quad (2)$$

where C_S represents the specific capacitance of the sample, assuming C_S = 0.040 mF cm^{-2} in 1 M KOH.[1]

Fabrication of Rechargeable Zinc-Air Batteries

A home-made cell was fabricated to measure the performance of the catalysts in rechargeable zinc-air batteries. A zinc plate with a thickness of 0.25 mm was used as the anode, and the gas diffusion layer (carbon paper) coated with the catalyst as the air cathode. The carbon paper with a geometric area of 1 cm^2 was used and 10 mg of the catalyst was loaded onto the paper. For the electrolyte, 0.2 M zinc acetate was dissolved in 6 M KOH to form zincate ($Zn(OH)_4^{2-}$) to ensure reversible zinc electrochemical reactions at the anode [4]. To prepare the catalyst slurry, 10 mg of HESOx/OLC or HESOx-550/OLC_{AT} was dissolved in 20 µL of water, 180 µL of isopropanol, and 20 µL of PTFE and stirred on a magnetic stirrer. The coated catalyst was oven-dried at 80 °C for 30 min. A rechargeable zinc-air battery with a mixture of Pt/C and IrO_2 (mass ratio of 1:1) was assembled in the same way for comparison. A Biologic VSP300 potentiostat was used to collect the reported data on rechargeable zinc-air batteries. The long-term stability was first conducted under shallow cycling conditions at 2 mA cm^{-2} with 30 min discharge and 30 min charge (1 h per cycle). The catalyst was then subjected to harsh cycling conditions (6 h per cycle then 12 h per cycle) to meet the minimum required depth of discharge.

Computational Studies

DFT calculations were conducted using the computing facilities at the Centre for High Performance Computing (CHPC, Cape Town, South Africa) using the BIOVIA Material Studio Suites and employing an adsorption locator tool module. Here, we designed and studied two model catalysts, HESOx/OLC and defective HESOx/OLC_{AT} utilizing spin-polarized density functional theory (DFT) calculations within the DMol3 module of Material Studio. Specifically, we focused on (311) surface of these spinel oxide catalysts sitting on OLC (002), as determined by XRD analysis. To model the defective HESOx/OLC_{AT}, some metal and oxygen atoms were knocked off from either the HESOx or the OLC or both (abbreviated as $HESOx_p/OLC_p$, $HESOx_p/OLC_d$, or $HESOx_d/OLC_d$ where the subscripts "p" and "d" mean pristine and defective, respectively). Supercells of 3x3 were modeled for the electrocatalysts. Material studio cleaning tool was used, followed by geometric relaxation calculations with a set threshold energy at 10^{-6} eV for convergence. The lowest adsorption distance was set at 5 Å. The adsorbates for the ORR and OER were chosen as O*, O_2, OH*, and OOH. Prior to the $Dmol^3$ simulation, the adsorption locator module was used to predict the preferred adsorption mechanistic models, in which the first model was found to be more stable.

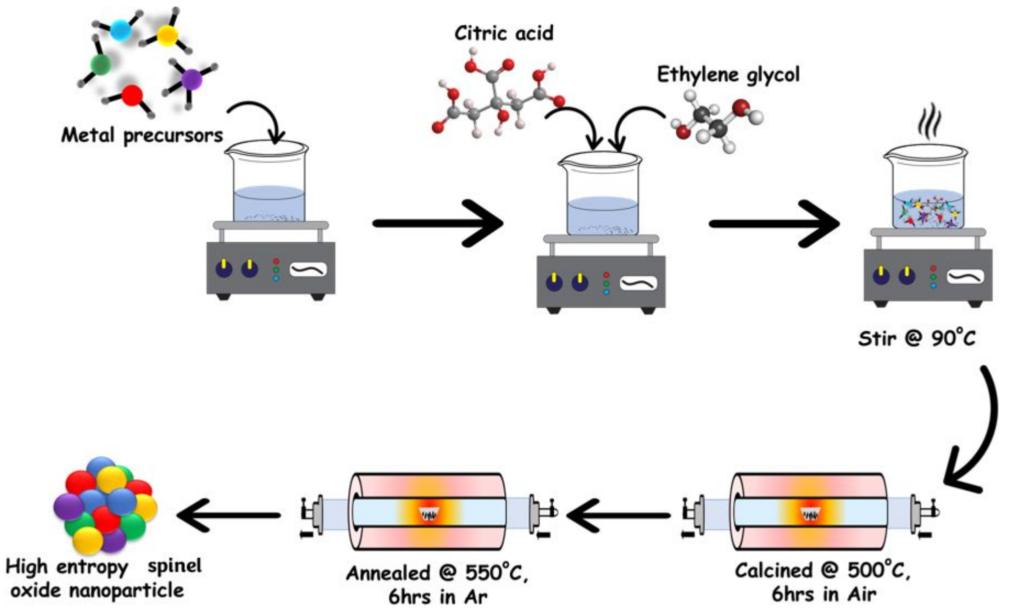

Figure S1. The schematic representation for the preparation of high entropy spinel oxides $(CoCuFeMnNi)_3O_4$.

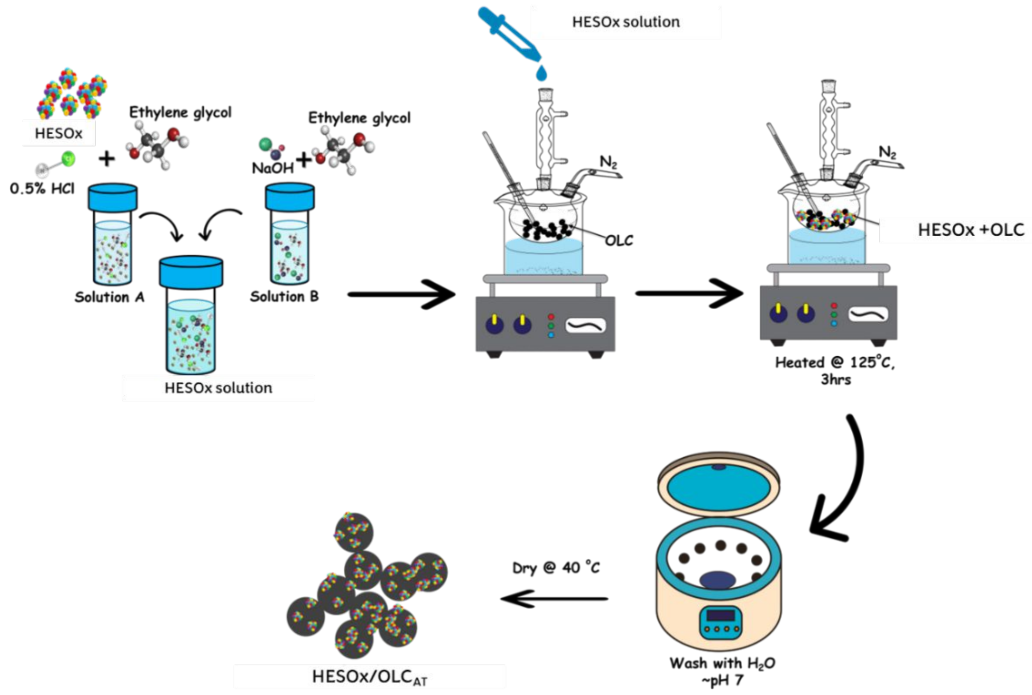

Figure S2. The schematic representation for loading HESOx onto onion-like carbon (OLC).

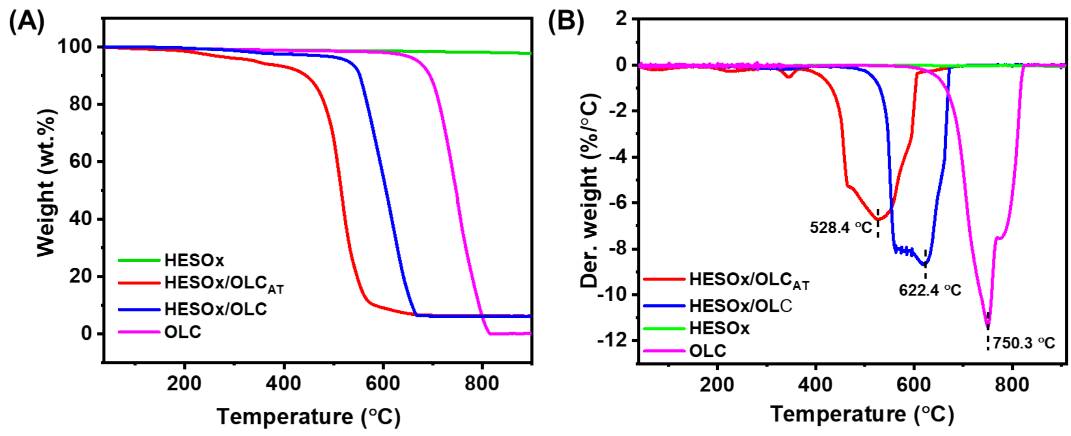

Figure S3: Thermogravimetric analysis of HESOx, HESOx/OLC$_{AT}$, HESOx/OLC, and OLC.

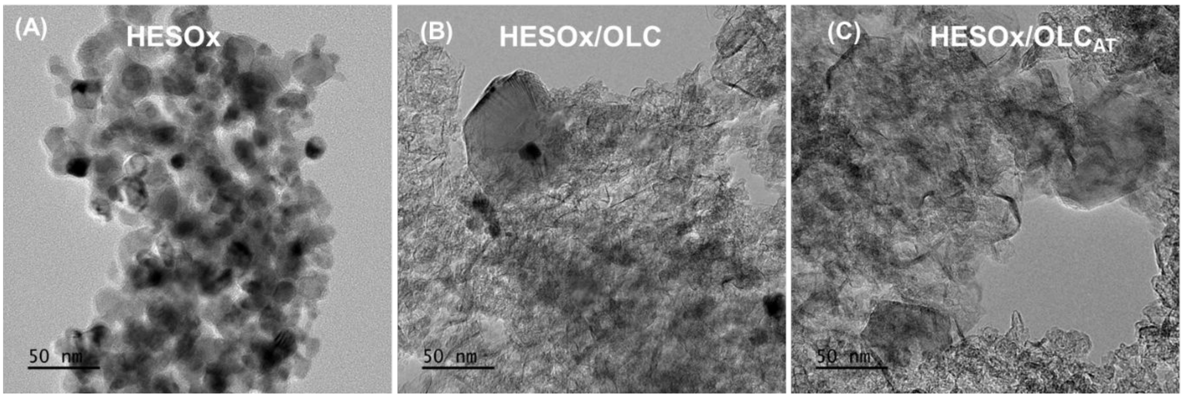

Figure S4. The HR-TEM images of (A) pristine HESOx, (B) HESOx/OLC, and (C) HESOx/OLC$_{AT}$.

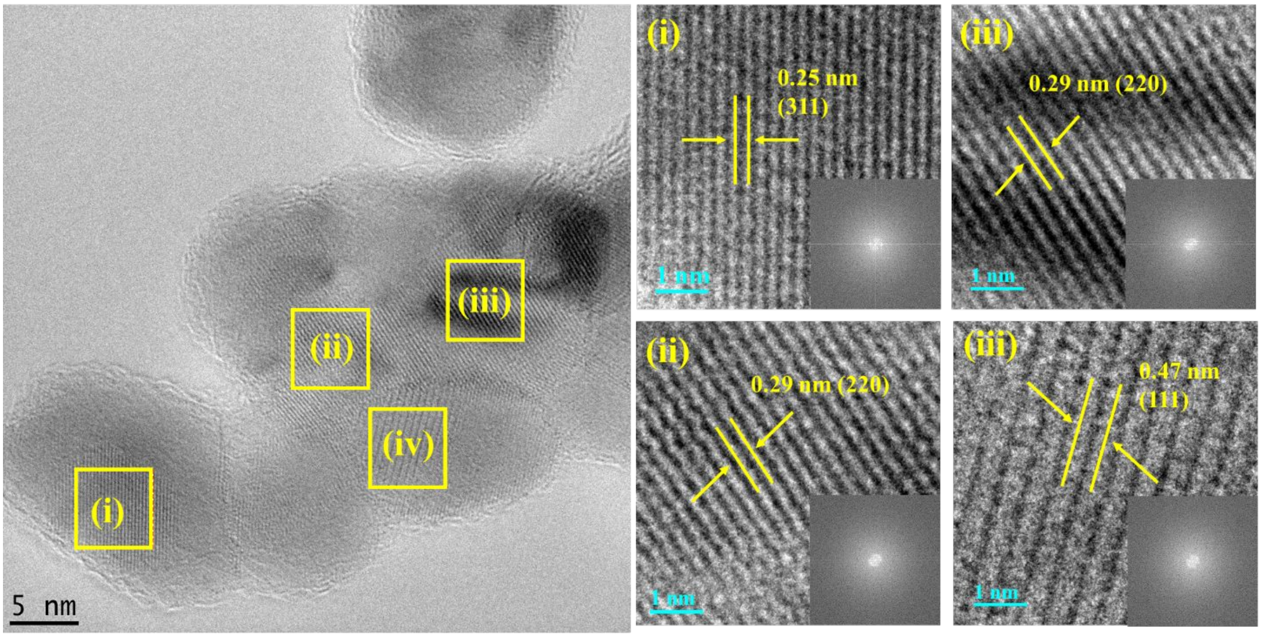

Figure S5. The HR-TEM images of pristine HESOx

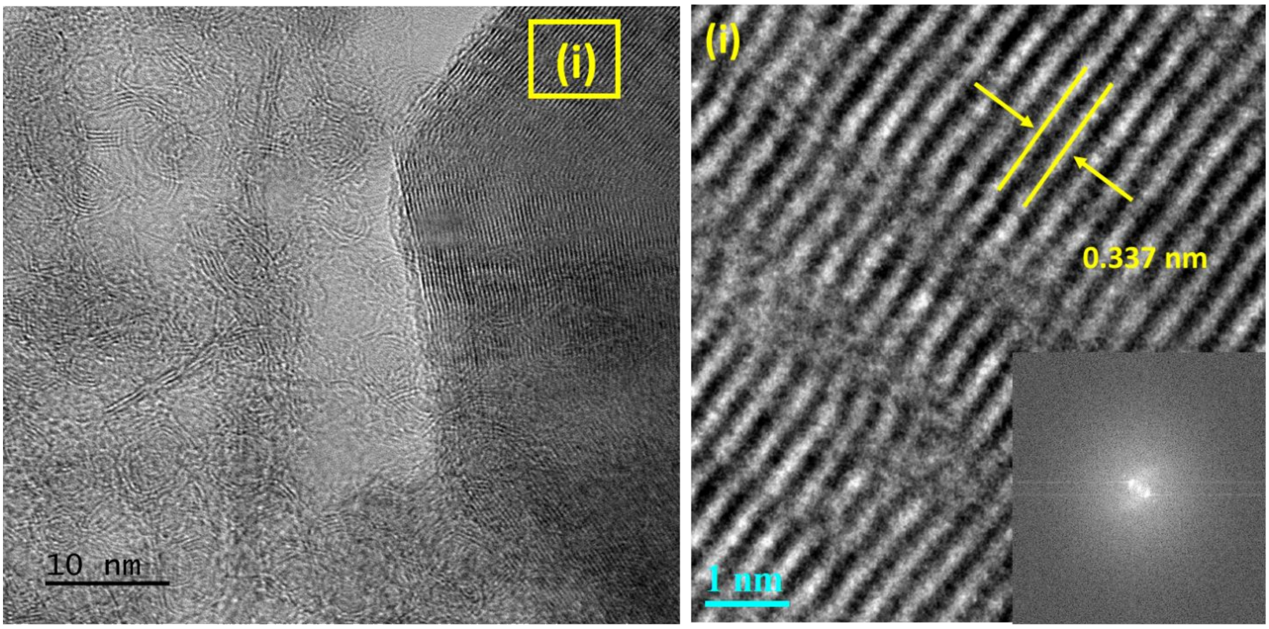

Figure S6. The HR-TEM image of HESOx/OLC

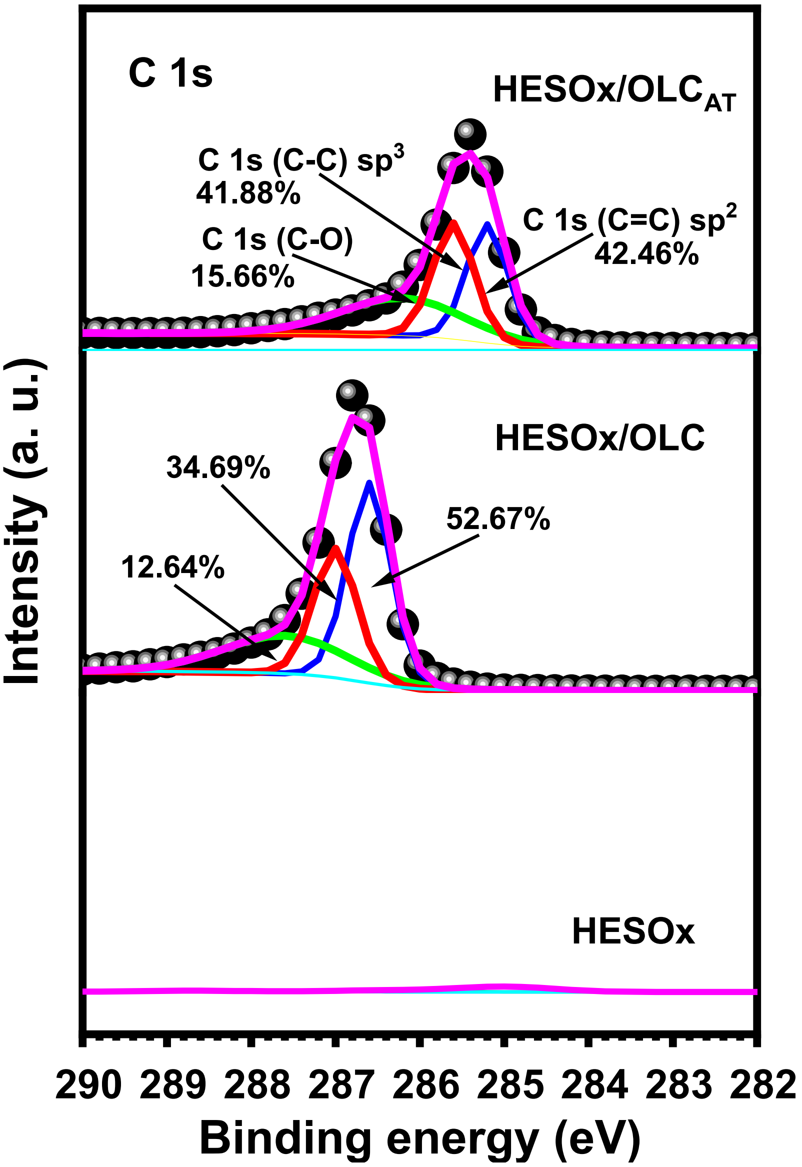

Figure S7. Deconvoluted XPS spectra of C1s of the pristine HESOx, HESOx/OLC and HESOx/OLC$_{AT}$.

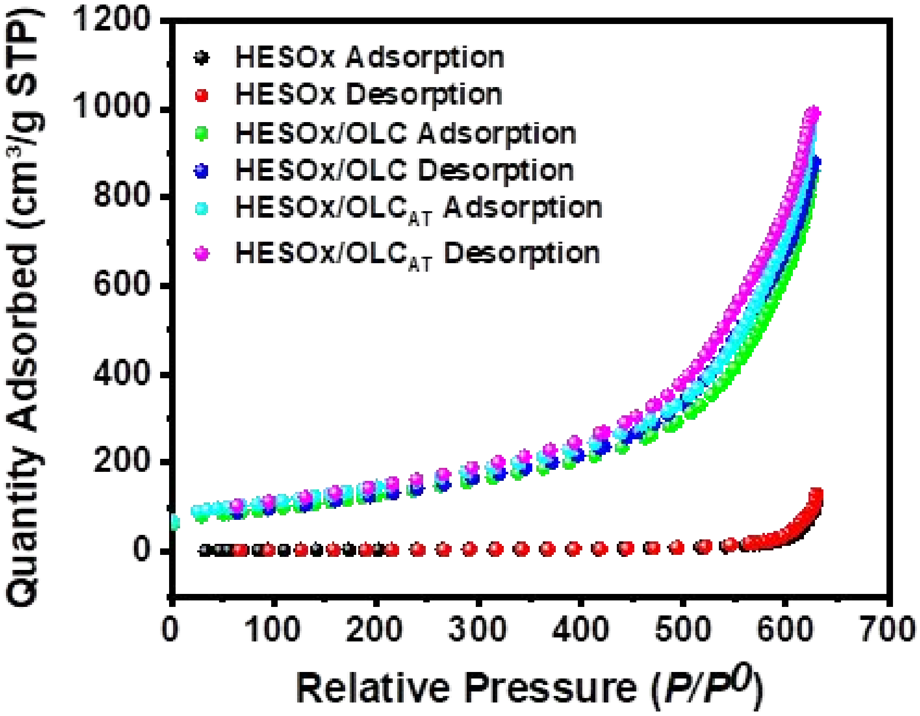

Figure S8. Nitrogen adsorption/desorption of the pristine HESOx, HESOx/OLC, and HESOx/OLC_{AT}.

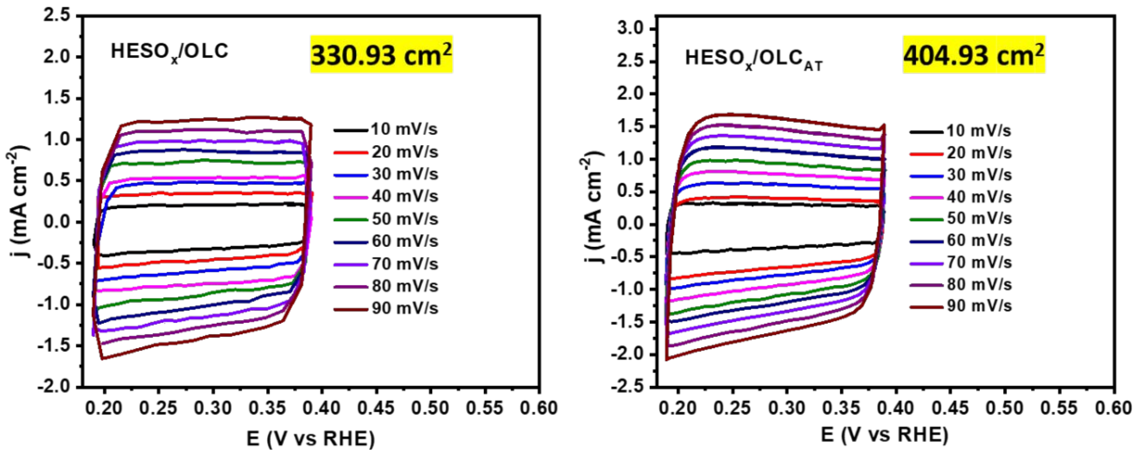

Figure S9. CV evolutions of HESOx/OLC and HESOx/OLC_{AT} at the capacitive region (0.18 – 0.38 V vs RHE) to determine the electrochemical active surface areas (ECSA cm^{-2}).

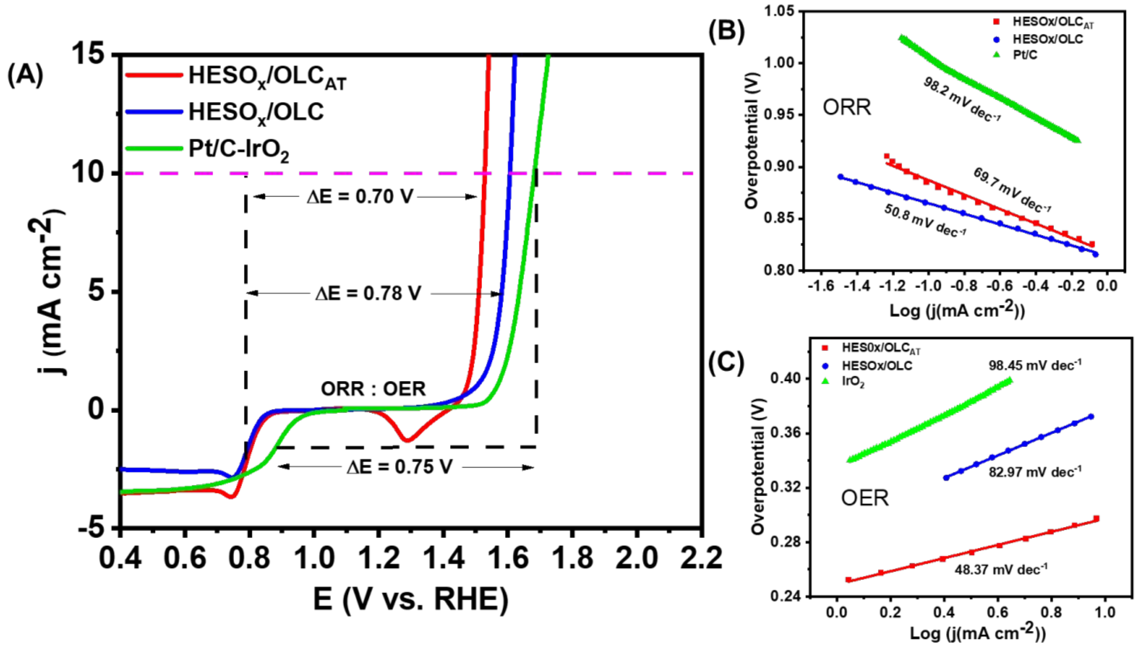

Figure S10. (A) ORR/OER bifunctional curves, and Tafel slopes for (B) ORR and (C) OER for all catalysts investigated in 1 M KOH.

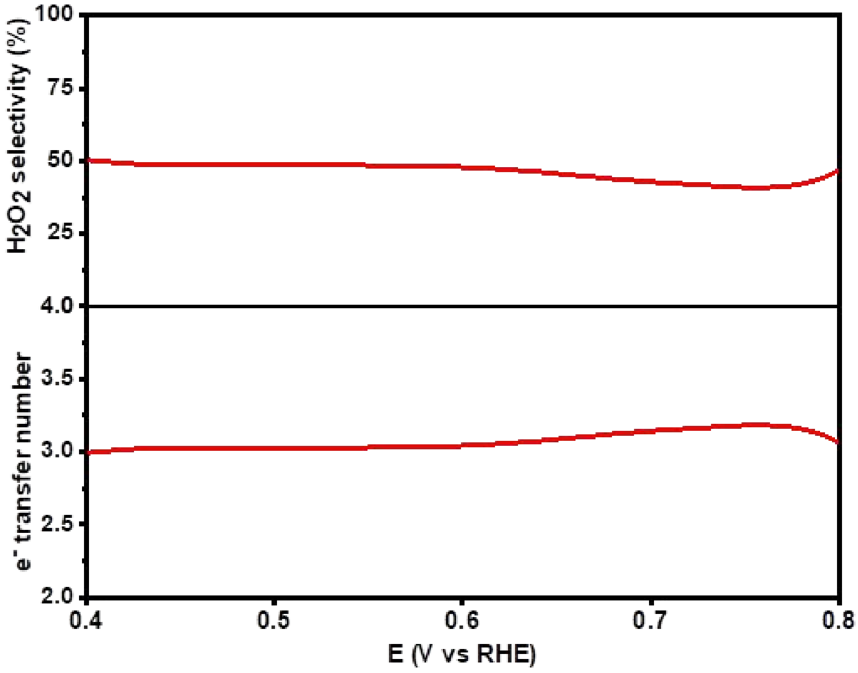

Figure S11. The electron transfer number and amount of hydrogen peroxide (H_2O_2) produced in an alkaline medium for $HESO_x/OLC_{AT}$.

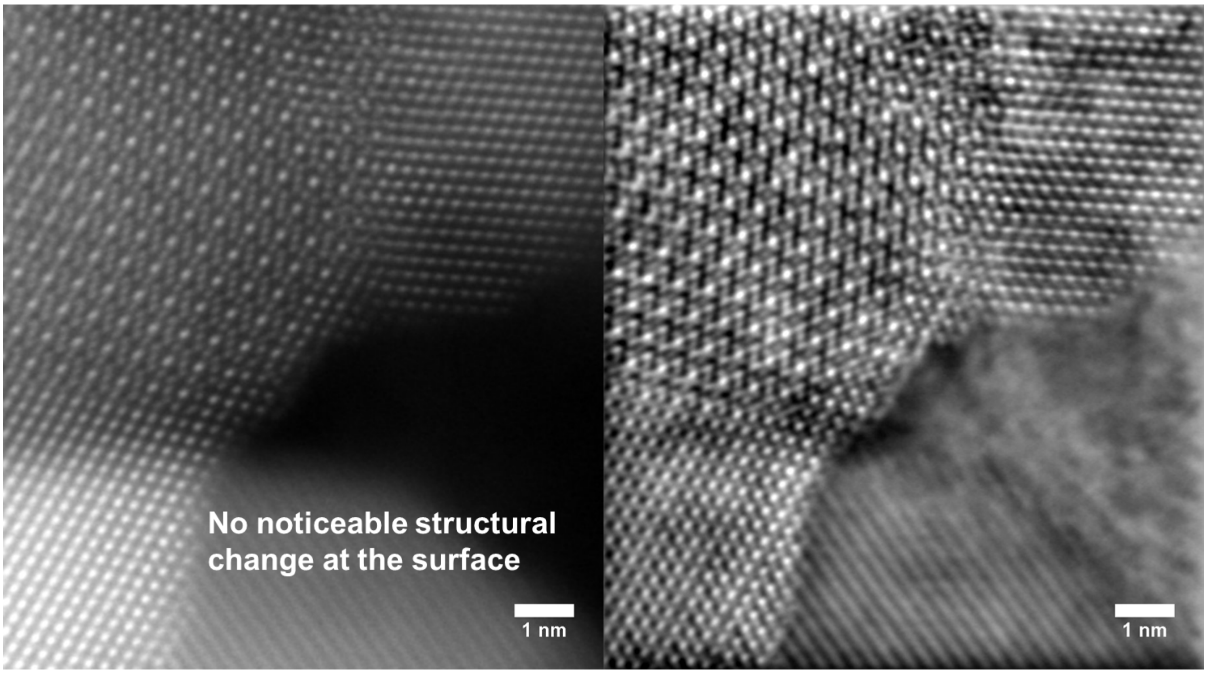

Figure S12. STEM of the HESOx/OLC$_{AT}$ at the end-of-life HESOx/OLC$_{AT}$ after ADTs (30k cycles) showing no noticeable structural change from its original inverse spinel structure.

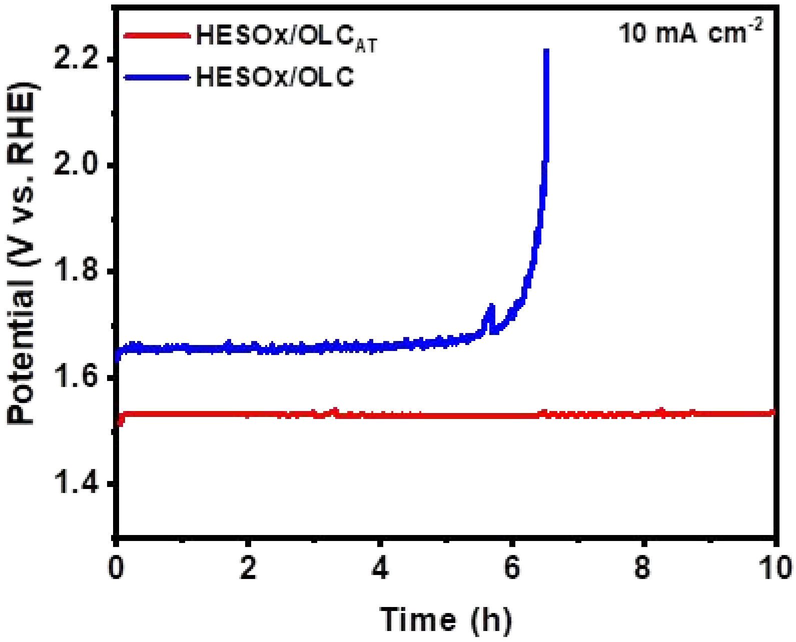

Figure S13. Stability test using chronoamperometric technique.

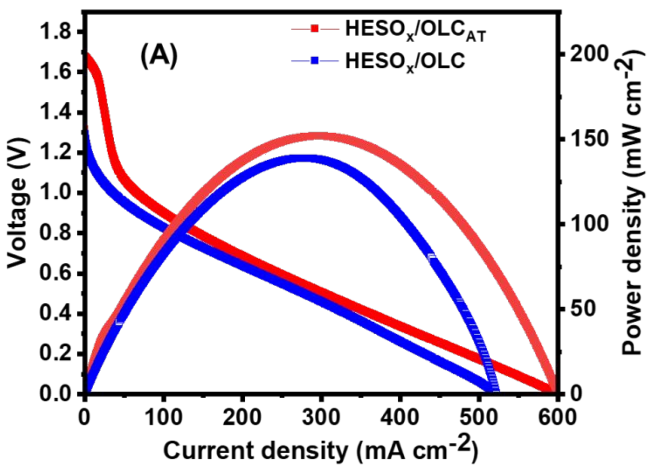

Figure S14. (A) Discharge polarization curves and corresponding power density curves of the ReZAB based on the HESOx/OLC and HESOx/OLC_{AT} from LSV, and (B) Typical discharge-charge polarization curves of the HESOx/OLC_{AT} based RZAB at a constant current density of 2 mA cm^{-2} between 5 and 300 cycles.

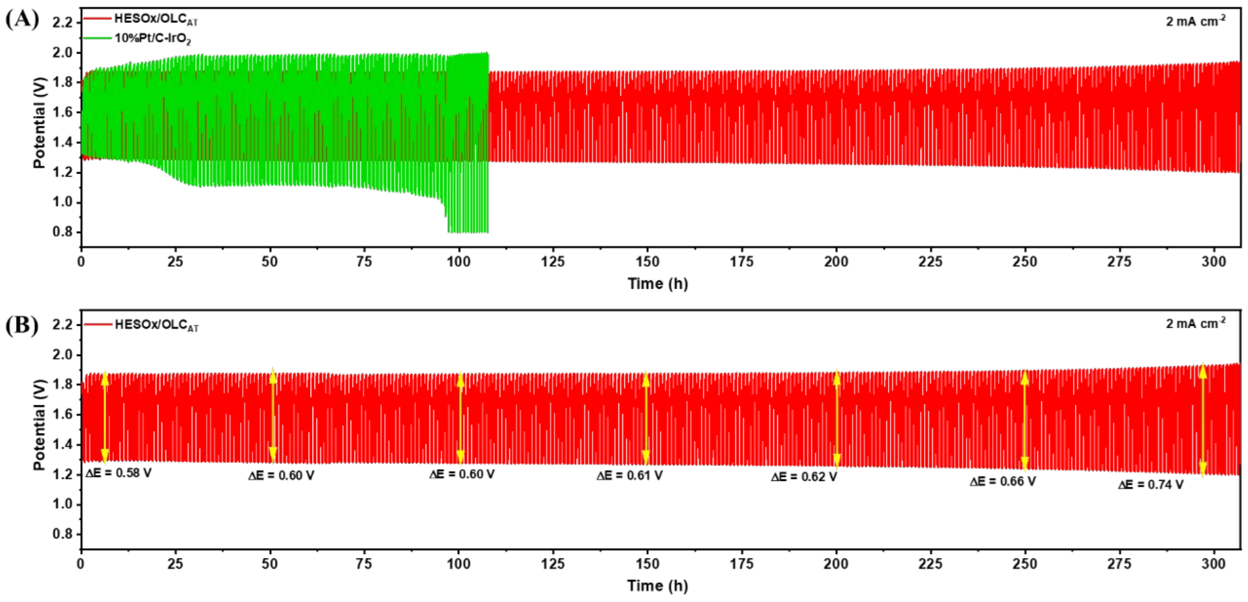

Figure S15. (A) Discharge-Charge curves of HESOx/OLC_{AT} and 10% Pt/C-IrO_2 at a current density of 2 mA cm^{-2} for 1 h per cycle.

Table S1: Brunauer-Emmett-Teller (BET) data for the HESOx materials

Catalyst	BET surface area (m^2/g)	Pore volume (cm^3/g)	Pore size (Å)
HESOx	14.83	0.0461	124.45
HESOx/OLC	387.97	0.987	101.79
HESOx/OLC_{AT}	434.21	1.11	100.09

Table S2. Summary of the d-band centers for ORR/OER intermediates.

**Catalyst**	**Intermediate**	**d-band centre (ε_d / eV)**
HESOx	O*	**-1.90804**
	O_2*	-1.90887
	OH*	**-1.90310**
	OOH*	-1.90365
$HESOx_p/OLC_p$	O*	**-3.32048**
	O_2*	-3.32098
	OH*	**-3.33420**
	OOH*	-3.32130
$HESOx_p/OLC_d$	O*	-3.32082
	O_2*	-3.32114
	OH*	**-3.32106**
	OOH*	-3.32136
$HESOx_d/OLC_d$	O*	**-3.25351**
	O_2*	-3.25370
	OH*	**-3.24195**
	OOH*	-3.24973

Table S3: Comparing the performance of HESOx/OLC_{AT} with medium- to high-entropy material-based air-breathing catalysts used for rechargeable zinc-air batteries. **NOTE:** Some of the unavailable area energy densities in the cited literature were calculated using the data reported in the articles.

**Medium- to High-Entropy Catalyst**	**Current Density ($mAcm^{-2}$)**	**Discharge Time**	**Discharge Voltage (V)**	**Areal Energy ($mWhcm^{-2}$)**	**Cycle life (h / cycle)**	**References**
HESOx/OLC_{AT}	10	6 h	~ 1.2	44 – 50	316 h (40)	This work
HESOx/OLC_{AT}	10	4 h	~ 1.2	37	40 h (7)	This work
HESOx/OLC_{AT}	10	3 h	~ 1.2	74	36 h (6)	This work
$Fe_{3\%}Co_{3\%}Ni_{9\%}$-NC1000	5	10 min	1.25	1.04	250 h (750 cycles)	Zhai *et al.* (2024)[2]
$CoMoO_4$-RuO_2	5	5 min	1.2	0.5	110 h	Chen *et al.* (2024)[3]
CoN/MnO@NC	10	10 min	1.25	2.08	480 h (720 cycles)	Niu *et al.* (2024)[4]
MnO-CeO_2@Cs + RuO_2	5	10 min	1.25	1.04	297 h	Wang *et al.* (2024)[5]
$Fe_6Ni_{20}Co_2Mn_2Cu_{1.5}$@rGO	10	30 min	1.1	5.5	300 h	Gao *et al.* (2024)[6]
CrMnFeCoNi	8	10 min	1.10	1.47	720 cycles	He *et al.* (2023)[7]
$Fe_{12}Ni_{23}Cr_{10}Co_{30}Mn_{25}$/CNT	5	7.5 min	1.20	0.75	256 h	Cao *et al.* (2023)[8]
FeCoNiMnCu-1000(1:1)	10	10 min	1.10	1.83	200 h/ 1005 cycles	Yao *et al.* (2023)[9]
Co-N-C@ CoNiFe-LDH	5	5 min	1.18	0.492	950h	Arafat *et al.* (2023)[10]

MoNiFe	10		1.20		450 cycles	Dang *et al.* et al. (2023)[11]
AlNiCoFeCrMoV-based HEO/CoNC	10	30 min	1.10	5.5	125 h/Cycles	Yu *et al.* (2023)[12]
(PtPdAuAgCuIrRu)clusters@ $(AlNiCoFeCrMoTi)_3O_4$	10	30 min	1.20	6.0	250 cycles	Jin *et al.* (2022)[13]
MnNiCuCoVFeMoPdPtAuRuIr/NG	2	30 min	1.25	1.25	300 h/ 300 cycles	Lin *et al.* (2022)[14]
AlNiCoRuMoCrFeTi	10	1 h	1.10	11.0	300 h/ 150 cycles	Jin *et al.* (2022)[15]
$Fe_4Co_1Ni_2$@hNCTs	10	10 min	1.14	1.19	310 cycles (110 h)	Tang *et al.* (2022)[16]
$Pd_{45}Pt_{44}Ni_{11}$ SpNSs/C	10	10 min	1.10	1.83	660 cycles (220 h)	Liu *et al.* (2022)[17]
Ox-MnCoNi-C	10	10 min	1.23	2.05	303 cycles (100 h)	Rui *et al.* (2022)[18]
FeCoNi@HNC	5	10 min	1.22	1.02	200 cycles	Liu *et al.* (2021)[19]
Mn-rich dodeca-alloy (np-12)	10	30 min	1.16	5.8	240 cycles	Yu *et al.* (2021)[20]
np−AlCoFeMoCr/Pt	20	30 min	1.12	11.2	50 cycles	Jin *et al.* (2021)[21]
MnCoNi-C-D	10	15 min	1.20	3.0	180 h	Wang *et al.* (2021)[22]
AlNiCoRuMo	10	30 min	1.20	6.0	80 h	Jin *et al.* (2020)[23]
AlFeCoNiCr	20	5 min	1.15	1.92	120 h/ 720 cycles	Fang *et al.* (2020)[24]
FeCoMoS@NG	2	5 min	1.20	0.2	70 h	Ramakrishnan *et al.* (2020)[25]
Co-Mn-Ni	10	5 min	1.23	1.025	100 cycles	Wang *et al.* (2019)[26]
FeCoNi-CNF	20	5 min	0.90	1.5	22 h	Li *et al.* (2019)[27]

FeCo/FeCoNi@NCNTs-HF	5	20 min	1.21	2.02	240 h (360 cycles)	Wang *et al.* (2019)[28]
$Ni_{46}Co_{40}Fe_{14}$ (C@ NCF)	50	6 h	1.25	375	8 cycles	Nam *et al.* . (2018)[29]
$M_{0.1}Ni_{0.9}Co_2O_4$ (M: Mn, Fe, Cu, Zn)	10	20 min	1.18	3.93	100 cycles	Lu *et al.* (2017)[30]
High-entropy prussian blue analogues (HEPBAs) (CuMnFeNiCo)	5	5 min	~ 1.2	0.5	40 h	Tanmathusorachai *et al.* (2024)[31]
FeCoNiPdWP (phosphides)	8	10 min	~ 1.1	1.47	700 h	He *et al.* (2024)[32]
(FeNiMnCuCr)3O4/rGO	10	5 min	~ 1.1	0.92	1162 h	Li *et al.* (2025)[33]
CoCuFeAgRu HEA	10	30 min	~ 1.0	5	40 h	Qiu *et al.* (2025)[34]

References

(1) McCrory, C. C.; Jung, S.; Peters, J. C.; Jaramillo, T. F. Benchmarking heterogeneous electrocatalysts for the oxygen evolution reaction. *Journal of the American Chemical Society* **2013**, *135* (45), 16977-16987.

(2) Zhai, W.; He, Y.; Duan, Y.-e.; Guo, S.; Chen, Y.; Dai, Z.; Liu, L.; Tan, Q. Densely populated trimetallic single-atoms for durable low-temperature flexible zinc-air batteries. *Applied Catalysis B: Environmental* **2024**, *342*, 123438.

(3) Chen, S.; Xu, J.; Chen, J.; Yao, Y.; Wang, Z.; Li, P.; Li, Y.; Wang, F. Ru doping induced interface engineering in flower-liked CoMoO4-RuO2 boosts oxygen electrocatalysis for rechargeable Zn-air battery. *Journal of Colloid and Interface Science* **2024**, *658*, 230-237.

(4) Niu, Y.; Jiang, G.; Gong, S.; Liu, X.; Shangguan, E.; Li, L.; Chen, Z. Engineering of heterointerface of ultrathin carbon nanosheet-supported CoN/MnO enhances oxygen electrocatalysis for rechargeable Zn–air batteries. *Journal of Colloid and Interface Science* **2024**, *656*, 346-357.

(5) Wang, L.; Hu, X.; Li, H.; Huang, Z.; Huang, J.; Isimjan, T. T.; Yang, X. Engineering built-in electric fields in oxygen-deficient MnO-CeO 2@ Cs catalysts: enhanced performance and kinetics for the oxygen reduction reaction in aqueous/flexible zinc–air batteries. *Green Chemistry* **2024**, *26* (4), 2011-2020.

(6) Gao, L.; Zhong, X.; Li, Z.; Hu, J.; Cui, S.; Wang, X.; Xu, B. A multi-layer reduced graphene oxide catalyst encapsulating a high-entropy alloy for rechargeable zinc-air batteries. *Chemical Communications* **2024**, *60* (10), 1269-1272.

(7) He, R.; Yang, L.; Zhang, Y.; Wang, X.; Lee, S.; Zhang, T.; Li, L.; Liang, Z.; Chen, J.; Li, J. A CrMnFeCoNi high entropy alloy boosting oxygen evolution/reduction reactions and zinc-air battery performance. *Energy Storage Materials* **2023**, *58*, 287-298.

(8) Cao, X.; Gao, Y.; Wang, Z.; Zeng, H.; Song, Y.; Tang, S.; Luo, L.; Gong, S. FeNiCrCoMn high-entropy alloy nanoparticles loaded on carbon nanotubes as bifunctional oxygen catalysts for rechargeable zinc-air batteries. *ACS Applied Materials & Interfaces* **2023**, *15* (27), 32365-32375.

(9) Yao, Y.; Li, Z.; Dou, Y.; Jiang, T.; Zou, J.; Lim, S. Y.; Norby, P.; Stamate, E.; Jensen, J. O.; Zhang, W. High entropy alloy nanoparticles encapsulated in graphitised hollow carbon tubes for oxygen reduction electrocatalysis. *Dalton Transactions* **2023**, *52* (13), 4142-4151.

(10) Arafat, Y.; Zhong, Y.; Azhar, M. R.; Asif, M.; Tadé, M. O.; Shao, Z. CoNiFe-layered double hydroxide decorated Co-N-C network as a robust bi-functional oxygen electrocatalyst for zinc-air batteries. *EcoMat* **2023**, *5* (10), e12394.

(11) Dang, L.; Zhang, K.; Wang, Q.; Xu, C.; Wang, S. Fe-Alloyed MoNi Nanohybrids as Oxygen Evolution Reaction/Oxygen Reduction Reaction Bifunctional Electrocatalyst for Rechargeable Zinc–Air Batteries. *physica status solidi (a)* **2023**, *220* (2), 2200581.

(12) Yu, T.; Xu, H.; Jin, Z.; Zhang, Y.; Qiu, H.-J. Noble metal-free high-entropy oxide/Co-NC bifunctional electrocatalyst enables highly reversible and durable Zn-air batteries. *Applied Surface Science* **2023**, *610*, 155624.

(13) Jin, Z.; Zhou, X.; Hu, Y.; Tang, X.; Hu, K.; Reddy, K. M.; Lin, X.; Qiu, H.-J. A fourteen-component high-entropy alloy@ oxide bifunctional electrocatalyst with a record-low Δ E of 0.61 V for highly reversible Zn–air batteries. *Chemical Science* **2022**, *13* (41), 12056-12064.

(14) Lin, X.; Hu, Y.; Hu, K.; Lin, X.; Xie, G.; Liu, X.; Reddy, K. M.; Qiu, H.-J. Inhibited surface diffusion of high-entropy nano-alloys for the preparation of 3D nanoporous graphene with high amounts of single atom dopants. *ACS Materials Letters* **2022**, *4* (5), 978-986.

(15) Jin, Z.; Lyu, J.; Hu, K.; Chen, Z.; Xie, G.; Liu, X.; Lin, X.; Qiu, H. J. Eight-component nanoporous high-entropy oxides with low Ru contents as high-performance bifunctional catalysts in Zn-air batteries. *Small* **2022**, *18* (12), 2107207.

(16) Tang, W.; He, J.; Teng, K.; Gao, L.; Qi, R.; Deng, Y.; Liu, R.; Li, A.; Fu, H.; Wang, C.-a. Toward highly efficient bifunctional electrocatalysts for zinc–air batteries: from theoretical prediction to a ternary FeCoNi design. *Nanoscale* **2022**, *14* (46), 17447-17459.

(17) Liu, K.; Huang, H.; Zhu, Y.; Wang, S.; Lyu, Z.; Han, X.; Kuang, Q.; Xie, S. Edge-segregated ternary Pd–Pt–Ni spiral nanosheets as high-performance bifunctional oxygen redox electrocatalysts for rechargeable zinc–air batteries. *Journal of Materials Chemistry A* **2022**, *10* (7), 3808-3817.

(18) Rui, C.; Zhang, T.; Jiang, Y.; Xie, D.; Li, M.; Lu, Q.; Bu, Y. Highly Efficient and Stable Bifunctional Electrocatalyst with Alloy/Oxide Heterostructures for a Rechargeable Zinc–Air Battery. *Energy & Fuels* **2022**, *36* (20), 12816-12825.

(19) Liu, J.; Luo, Z.; Zhang, X.; Zheng, H.; Peng, L.; Qian, D.; Jia, C.; Sun-Waterhouse, D.; Waterhouse, G. I. FeCoNi nanoalloys embedded in hierarchical N-rich carbon matrix with enhanced oxygen electrocatalysis for rechargeable Zn-air batteries. *Journal of Materials Chemistry A* **2021**, *9* (48), 27701-27708.

(20) Yu, T.; Zhang, Y.; Hu, Y.; Hu, K.; Lin, X.; Xie, G.; Liu, X.; Reddy, K. M.; Ito, Y.; Qiu, H.-J. Twelve-component free-standing nanoporous high-entropy alloys for multifunctional electrocatalysis. *ACS Materials Letters* **2021**, *4* (1), 181-189.

(21) Jin, Z.; Lyu, J.; Zhao, Y.-L.; Li, H.; Chen, Z.; Lin, X.; Xie, G.; Liu, X.; Kai, J.-J.; Qiu, H.-J. Top–down synthesis of noble metal particles on high-entropy oxide supports for electrocatalysis. *Chemistry of Materials* **2021**, *33* (5), 1771-1780.

(22) Wang, X.; Zhang, J.; Ma, D.; Feng, X.; Wang, L.; Wang, B. Metal–organic framework-derived trimetallic nanocomposites as efficient bifunctional oxygen catalysts for zinc–air batteries. *ACS Applied Materials & Interfaces* **2021**, *13* (28), 33209-33217.

(23) Jin, Z.; Lyu, J.; Zhao, Y.-L.; Li, H.; Lin, X.; Xie, G.; Liu, X.; Kai, J.-J.; Qiu, H.-J. Rugged high-entropy alloy nanowires with in situ formed surface spinel oxide as highly stable electrocatalyst in Zn–air batteries. *ACS Materials Letters* **2020**, *2* (12), 1698-1706.

(24) Fang, G.; Gao, J.; Lv, J.; Jia, H.; Li, H.; Liu, W.; Xie, G.; Chen, Z.; Huang, Y.; Yuan, Q. Multi-component nanoporous alloy/(oxy) hydroxide for bifunctional oxygen electrocatalysis and rechargeable Zn-air batteries. *Applied Catalysis B: Environmental* **2020**, *268*, 118431.

(25) Ramakrishnan, S.; Balamurugan, J.; Vinothkannan, M.; Kim, A. R.; Sengodan, S.; Yoo, D. J. Nitrogen-doped graphene encapsulated FeCoMoS nanoparticles as advanced trifunctional catalyst for water splitting devices and zinc–air batteries. *Applied Catalysis B: Environmental* **2020**, *279*, 119381.

(26) Wang, Q.; Xue, Y.; Sun, S.; Yan, S.; Miao, H.; Liu, Z. Facile synthesis of ternary spinel Co–Mn–Ni nanorods as efficient bi-functional oxygen catalysts for rechargeable zinc-air batteries. *Journal of Power Sources* **2019**, *435*, 226761.

(27) Li, C.; Zhang, Z.; Wu, M.; Liu, R. FeCoNi ternary alloy embedded mesoporous carbon nanofiber: an efficient oxygen evolution catalyst for rechargeable zinc-air battery. *Materials Letters* **2019**, *238*, 138-142.

(28) Wang, Z.; Ang, J.; Zhang, B.; Zhang, Y.; Ma, X. Y. D.; Yan, T.; Liu, J.; Che, B.; Huang, Y.; Lu, X. FeCo/FeCoNi/N-doped carbon nanotubes grafted polyhedron-derived hybrid fibers as bifunctional oxygen electrocatalysts for durable rechargeable zinc–air battery. *Applied Catalysis B: Environmental* **2019**, *254*, 26-36.

(29) Nam, G.; Son, Y.; Park, S. O.; Jeon, W. C.; Jang, H.; Park, J.; Chae, S.; Yoo, Y.; Ryu, J.; Kim, M. G. A Ternary Ni46Co40Fe14 Nanoalloy-Based Oxygen Electrocatalyst for Highly Efficient Rechargeable Zinc–Air Batteries. *Advanced Materials* **2018**, *30* (46), 1803372.

(30) Lu, Y.-T.; Chien, Y.-J.; Liu, C.-F.; You, T.-H.; Hu, C.-C. Active site-engineered bifunctional electrocatalysts of ternary spinel oxides, M 0.1 Ni 0.9 Co 2 O 4 (M: Mn, Fe, Cu, Zn) for the air electrode of rechargeable zinc–air batteries. *Journal of Materials Chemistry A* **2017**, *5* (39), 21016-21026.

(31) Tanmathusorachai, W.; Aulia, S.; Rinawati, M.; Chang, L.-Y.; Chang, C.-Y.; Huang, W.-H.; Lin, M.-H.; Su, W.-N.; Yuliarto, B.; Yeh, M.-H. High-Entropy Prussian Blue Analogue Derived Heterostructure Nanoparticles as Bifunctional Oxygen Conversion Electrocatalysts for the Rechargeable Zinc–Air Battery. *ACS Applied Materials & Interfaces* **2024**, *16* (45), 62022-62032.

(32) He, R.; Wang, S.; Yang, L.; Horta, S.; Ding, Y.; Di, C.; Zhang, X.; Xu, Y.; Ibáñez, M.; Zhou, Y. Active site switching on high entropy phosphides as bifunctional oxygen electrocatalysts for rechargeable/robust Zn–air battery. *Energy & Environmental Science* **2024**, *17* (19), 7193-7208.

(33) Li, W.; Wang, Y.; Xu, N.; Li, Y. High-entropy spinel oxides as efficient ORR catalysts towards enhanced kinetics for zinc-air batteries. *Journal of Energy Storage* **2025**, *123*, 116784.

(34) Qiu, Z.; Guo, X.; Cao, S.; Du, M.; Wang, Q.; Pi, Y.; Pang, H. High-Entropy Ag− Ru-Based Electrocatalysts with Dual-Active-Center for Highly Stable Ultra-Low-Temperature Zinc-Air Batteries. *Angewandte Chemie International Edition* **2025**, *64* (3), e202415216.
